# Influential Spanish Politicians’ Discourse of Climate Change on Twitter: A Corpus-Assisted Discourse Study

**DOI:** 10.1007/s41701-023-00140-3

**Published:** 2023-04-29

**Authors:** Mai Osama Ghoraba

**Affiliations:** Faculty of Languages, Spanish Department, Ain Shams University, Cairo, Egypt

**Keywords:** Corpus linguistics, Critical discourse studies, Ecolinguistics, Keywords analysis, Climate change, Framing

## Abstract

This piece of research explores language use in a sample of unprecedentedly studied discourse which is that of climate change communication by influential Spanish politicians via Twitter. For that purpose, we created a specialized corpus composed of tweets tackling climate change that were posted by influential Spanish politicians during the past decade. Our aim was to reveal prominent linguistic patterns that are susceptible of conveying a specific worldview (i.e.: the wording of reality) of climate change to Twitter users. Our analysis started with keywords analysis in order to gather quantitative data about the lexical choices deployed in our corpus, then by means of qualitative analysis based on semantic classification of keywords and the examination of their concordances we were able to point out distinctive features of our corpus’ discourse. Our results have revealed the prevalence of specific linguistic patterns, metaphors and frames that contribute to create a narrative of climate change as a villain and the human race, specifically political leaders, as the saviour.

## Introduction

**Fig. 1:**
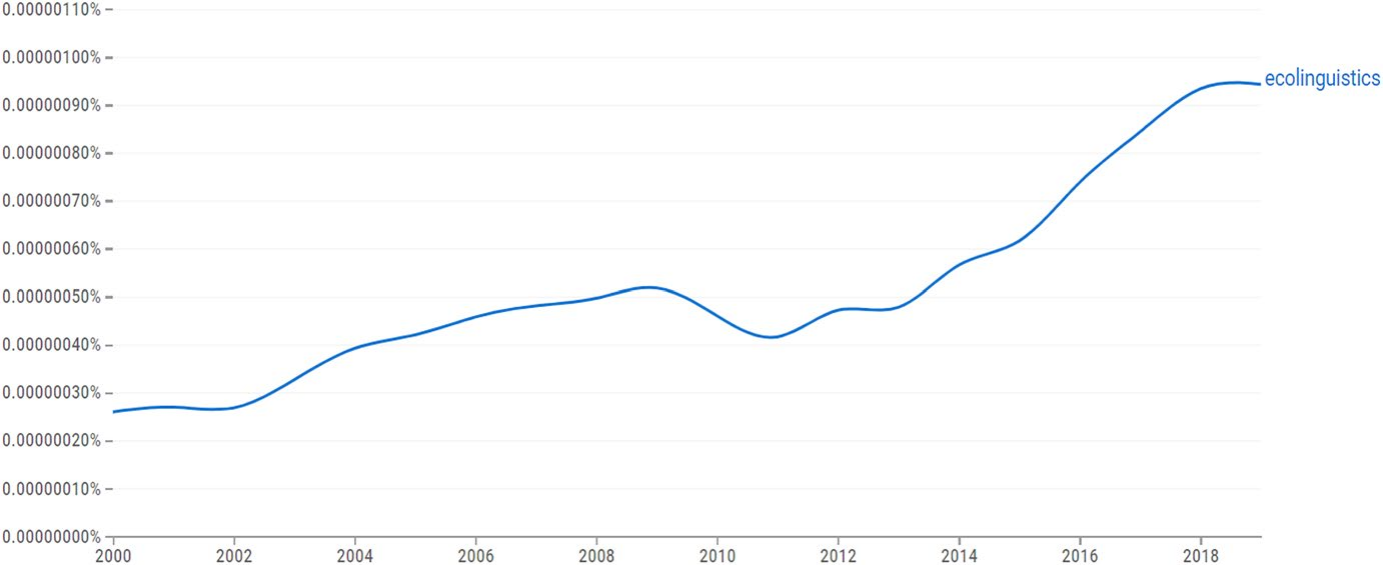
Frequency of the word “ecolinguistics” extracted from Google N gram Viewer, 2000 to 2019

In 2001, Halliday demonstrated how linguistic analysis plays a crucial role in finding ‘the key’ to environmental problems (e.g.: pollution and climate). In his article (Halliday, 2001), he also encouraged researchers to uncover worldviews enclosed in the linguistic choices deployed in planning and policy-making. Since then, there has been an increasing academic interest in (critical) ecolinguistics as evidenced in the following chart extracted from Google N gram viewer (Fig. 1).

Unfortunately, climate change communication in Spanish has not been getting equal scholarly attention. Previous studies on the construction of climate change perception in the Spanish media are scarce in quantity, in comparison with those carried out in other EU countries. It is no coincidence that Chávez (2013) and Hernández et al. (2016) pick the term “silence” to refer to poor media coverage of global warming or climate change in Spain.

Erviti and León (2017) performed a relatively recent review of the history of climate change communication in Spain since its beginnings in 1976 till the first couple of decades of the 21st century. Two of their conclusions are of special importance to the build-up of the current paper as they shed light on: (1) the lack of any academic coverage of climate change communication via social media platforms in Spain, and (2) the construction of climate change in the Spanish media as a ‘remote phenomenon (…) in contrast with economic problems’ and essentially as a ‘political issue’ (Erviti & León, 2017: 25). In what follows, we highlight the most relevant findings of previous academic studies concerning climate change communication in Spain.

### Literature Review

Through their content analysis of Spanish newspapers’ pieces covering the issue of climate change during 2005/2006 and 2011, Fernández-Reyes et al. (2015) revealed that pieces featuring “climate change”, “global warming” or “greenhouse effect” usually increase around UN Climate Change Conferences (COP). However, they also detected an inversely proportional relationship between publishing about climate change and the increased awareness and/or certainty about its consequences among the public. Thus, their findings suggest a possible manipulation of media coverage of climate change in order to shift the public’s attention away from the issue (Fernández-Reyes et al., 2015: 136).

Similar conclusions and recommendations can be found in the few academic studies performed before 2011.^1^ For instance, Fernández-Reyes (2010: 12) highlighted the importance of digital communication about global warming as an alternative to the controlled discourse of mass-media productions which were termed by “*oligopolios mediáticos*/media oligopoly”.

In fact, the majority of academic production on the Spanish worldview of climate change belongs to the social sciences domain as “[r]esearch has primarily focused on public perception and media coverage of climate change” (Erviti & León, 2017: 2). Only a couple of studies authored by Castilla et al. (2013) and Fernández-Vázquez and Sancho-Rodríguez (2020) were found to tackle prevalent frames in climate change discourse in printed media, in Spain. This later piece of research stands out as the first Spanish academic work applying critical discourse analysis to the study of Spanish global corporates’ discourse about climate change. The paper demonstrates the prevalence of the technology frame as the solution to mitigate the effects of climate change in IBEX 35 Spanish corporates’ discourse, as Fernández-Vázquez and Sancho-Rodríguez (2020: 12) explicitly state that: “in the IBEX 35 narratives the focus is on reducing the worst effects of climate change, but without taking any extreme action that could involve a variation in current developmental models”.

Therefore, this paper of exploratory-descriptive approach aims to fill current gap in climate change construction and portrayal via social media platforms in Spain. For practical reasons, we had to limit our analysis to the scrutiny of one register of social media discourse on climate change in Spain, which is that of influential Spanish politicians’ tweets about climate change or global warming. The following section presents the motives of selection of this particular text-variety to conform the corpus of the present study.

### Twitter and Political Discourse

Twitter was chosen among other social media platforms (e.g., *Facebook*, *YouTube*, etc.) to collect Spanish politicians’ online discourse about climate change, because it has become an indispensable political tool and the preferred medium for politicians to engage with the public (Carrasco-Polaino et al., 2018; Redek & Godnov, 2018).

In fact, the ‘register’^2^ of political discourse enacted via Twitter has gained wide scholarly attention during the past decade^3^ in the critical discourse studies’ (CDS) domain. The singularity of Twitter as a political tool for self-representation (i.e., identities’ enactment) and creating engagement with the public has been pointed out by various linguists who describe ‘microblogging’ as: a “playing field between well-organized groups with plentiful resources and nascent or marginal groups looking to influence the policymaking process” (Gupta et al., 2018); and as a “rare opportunity” (Michele Zappavigna, 2017: 216) for linguists to uncover mass social practices reflecting communal set of shared values or opinions. Thus, the use of Twitter in the political arena is viewed as a tactic to influence the perception of the public and to maintain the hegemony of the powerful ‘elite’, given the fact that “elite users, comprising less than 0.05% of the user population, attract almost 50% of all attention within Twitter” (Wu et al., 2011: 5).

Currently, political tweeting about climate change in Spain remains an understudied area. Only recently, few studies have approached this discourse from different perspectives (e.g.: Carrasco-Polaino et al., 2022; Rodrigo-Cano, 2020; Smolak Lozano & Nakayama, 2022), nevertheless, to our knowledge, no previous linguistic research has yet studied climate change or global warming tweets posted by Spanish politicians.

Before presenting the theoretical foundations upon which this study relies, it is necessary to shed light on the genre of political discourse, since our corpus’ texts are classified as pertaining to this genre. Within the CDS domain, political discourse is viewed as the use of language to achieve a convincing representation of a “given view of reality” (Filardo-Llamas & Boyd, 2018: 318). More precisely, the language of political discourse is a construct of linguistic patterns and devices—across all linguistic levels from lexis to pragmatics—employed to persuade with a particular “perspective” in a given “context” (Filardo-Llamas & Boyd, 2018; van Dijk, 1990; Wilson, 2005). That is why, political communication has been viewed as an act of storytelling or a narrative (Jones et al., 2014; Mottier, 2008; De Fina, 2018). At the heart of political discourse, ideological representation and narration lies the concept of metaphor, which is defined as: “a device for seeing something in terms of something else” (Burke, 1941: 421).

In this respect, Chilton and Schäffner (2002: 29) explain that:

Metaphor can provide a conceptual structure for a systematized ideology that is expressed in many texts and much talk. It provides intertextual coherence (…) Lexical items usually regarded as opaque, unanalyzable, and conventionalized, frequently turn out to have conceptual coherence through a common underlying metaphorical schema.

Therefore, it is not through the analysis of metaphors in a given text that we unravel latent mental models in political discourse, but it is by means of the analysis of lexical and semantic configuration of a text that we reveal “the various metaphorical instantiations of a common underlying conceptual metaphor” (Dirven et al., 2003: 7).

Taking all previous points into consideration, we have opted to limit our analysis to the lexical and semantic patterning of the present corpus’ discourse. In the following section, we present the theoretical and methodological approaches that lay the foundation for our analysis and results.

### Theoretical Underpinnings

This paper falls within critical discourse studies (CDS) which constitutes a broad research domain comprising a wide range of different methodologies and analytical frameworks, often of interdisciplinary nature, in order to provide a flexible toolkit for researchers to examine almost all kinds of discourse (e.g., textual, oral, visual). For instance, Twitter as a corpus has been approached by various methods and analytical frameworks within the realm of CDS; such as: appraisal theory by Martin and White (2005) in Zappavigna (2012), Narrative Policy Framework (NPF) developed by McBeth et al. (2014) in Gupta et al. (2018) and corpus linguistics methods (CorpLing) in Baker & McEnery (2015), to name a few.

The present study utilizes CorpLing tools in its methodological design, given its previously demonstrated efficiency in exploratory descriptive studies of textual corpora (e.g.: Baker, 2009; Stubbs, 2005). Despite the fact that the notion of “register” is central to this paper, the analysis performed in this study cannot be described as a ‘quantitative register analysis’ because of the critical dimension enclosed within the qualitative analysis (see Sect. 2). Thus, the present study’s objective is not to merely identify distinctive lexical and semantic features of the collected corpus representing a particular register, as it also attempts to explain and interpret the prominence of those distinctive features by drawing upon insights collected from pertinent analytical frameworks, following in that Baker and McEnery (2015) in their analysis of the *Benefits Street* corpus.

The final goal of the current study is to detect possible pervasive framings of climate change in Spanish politicians’ discourse on Twitter. The premise upon which our analysis is based, is that the selection of particular lexical items over others is driven by a specific worldview or an attitude (van Dijk, 1995). In their study of climate change discourse, Fløttum & Gjerstad (2017: 2) define framing as: “the process which implies a strategic selection (conscious or not) of language features for a particular purpose”. On her part, Moser (2010: 39) identifies a set of features that constitute a frame in climate change communication, stating that: “Frames are triggered by words, imagery, symbols, and non-verbal cues such as messengers, music, tone of voice, and gestures”. In fact, climate change communication, as a subset of environmental discourse, has been academically associated with the framing system as evidenced in Fernández-Vázquez and Sancho-Rodríguez (2020) and Dahl and Fløttum (2019), among others.

The reason behind this association is explained by Arran Stibbe, founder of the International Ecolinguistics Association, who argues that:

Ecolinguistics can explore the more general patterns of language that influence how people both think about, and treat, the world. It can investigate the stories we live by—mental models that influence behaviour and lie at the heart of the ecological challenges we are facing. (Stibbe, 2015: 1–2)

To recapitulate, the core of our analysis is statistically salient lexical patterns which are susceptible of conveying a particular worldview—taking into consideration the distinction between the reality (the world) and the wording of the reality (through language use). Then, it is through qualitative analysis of these salient lexical patterns use in context that recurrent themes are revealed, and as a result, the frames within which Spanish politicians tend to portray climate change on Twitter.

### Climate Change Situation in Spain

According to the latest briefing published by the EPRS about Climate action in Spain (Simões & Victoria, 2021), Spain has achieved the reduction of greenhouse gas (GHG) emissions by 27% in the period between 2017—following the participation of Spain in the Paris Agreement in 2016—and 2019, surpassing the European average of GHG emissions reduction. The report also specifies that the two largest sectors emitting GHG in Spain are: transport sector (27%) and energy industry sector (16%).

Despite that, the study issued by the Spanish Climate Change Office (Sanz & Galán, 2020) has warned of multiple high risks of climate change impact in Spain like: the rise of ocean temperature exceeding current low gas emission scenarios by two to four times and the reduction of water resources^4^, which will negatively impact energy production and the entire economic sector.

Given that the climate change impact in Spain started to be noticed around the seventies of the past decade, the government of Spain approved the Law 22/1988, Of July 28th, Of Coasts which has been subjected to many modification until it was changed into the Law 2/2013 of the 29th of May on the protection and sustainable use of coastal areas, according the MITECO (*Ministerio de la Transición Ecológica y el Reto Demográfico*) website (https://www.miteco.gob.es/). However, from 1988 to 2016, Spanish governmental legislations and action plans have failed to achieve favourable outcomes as reported by *The European Climate Adaptation Platform*:

Over the period 1980–2016, Spain was the fifth EU country with the highest economic losses in absolute terms caused by climate-related events, and increasing risks of droughts, biodiversity loss, forest fires, coastal flooding and heatwaves are among the critical ones and rank Spain in the top 3 most vulnerable EU Member States. (“Spain”, Climate-ADAPT, 2021)

In 2019, the COP25 was celebrated in Madrid, however, it was considered a failure in terms of the attempt to achieve significant reduction of global carbon emissions (Newell & Taylor, 2020). In addition to that, the report of the Spanish Climate Change Office (Sanz & Galán, 2020) also urged the Spanish government to establish more efficient adaptation policies to climate change impact in Spain as it concluded that climate change is already aggravated in Spain and that it will keep getting worse in the future, due to the lack of proper adaptation policies (Sanz & Galán, 2020).

As a result, Sánchez’s government approved the new Law 7/23, of May 20th, on Climate Change and Ecological Transition which has established the Spanish National Climate Change Adaptation Plan 2021–2030 (PNACC) (https://www.boe.es/).

## Materials and Methods

This section begins with the presentation of the study’s corpus design and creation, then we describe the methods followed to arrive at our study’s results. In order to build a representative and balanced corpus of influential Spanish politicians’ tweeting about the climate, we set the following inclusion criteria: (1) the most influential Spanish politicians according to recent statistics, (2) Spanish politicians who have verified accounts on Twitter, (3) Spanish politicians who post frequently about “climate change” or “global warming” (> 3 tweets), and (4) Spanish politicians whose posts achieve high engagement (scoring > 50 on *SparkToro* scale^5^).

Accordingly, the latest edition of the list of the most influential 50 political leaders in Spain for 2022, published by the Marqués de Oliva Foundation in collaboration with *Merca2* online periodical, was adopted for the purpose of selecting the subjects whose tweets would conform the corpus of the present study.^6^ Previous editions of this list have already been used in recent academic research papers like that authored by García-Santamaría et al. (2020). After the exclusion of those leaders who do not meet the inclusion criteria, the following list (Table 1) of the most influential Spanish politicians who tweet frequently about Climate change/global warming was obtained:

**Table 1:** List of included influential Spanish politicians

Name	Twitter account	*SparkToro* score
1. Alberto Garzón	@agarzon	99
2. Pedro Sánchez	@sanchezcastejon	99
3. Iñigo Errejón	@ierrejon	98
4. Irene Montero	@IreneMontero	96
5. Pablo Casado	@pablocasado_	96
6. Inés Arrimadas	@InesArrimadas	95
7. Yolanda Díaz Pérez	@Yolanda_Diaz_	95
8. Ada Colau	@AdaColau	91
9. José Luis Ábalos	@abalosmeco	89
10. Salvador Illa Roca	@salvadorilla	87
11. Begoña Villacís Sánchez	@begonavillacis	86
12. Juan Manuel Moreno	@JuanMa_Moreno	85
13. Ximo Puig	@ximopuig	83
14. Guillermo Fernández Vara	@GFVara	83
15. Unai Sordo	@UnaiSordo	76
16. Miguel Ángel Revilla	@RevillaMiguelA	75
17. Emiliano García-Page	@garciapage	69
18. Alberto Núñez Feijóo	@FeijooGalicia	66
19. José Manuel Albares Bueno	@jmalbares	64
20. Alfonso Fernando Mañueco	@alferma1	61
21. Meritxell Batet Lamaña	@meritxell_batet	59
22. Reyes Maroto	@MarotoReyes	57
23. Iñigo Urkullu	@iurkullu	57

The following step was to extract the above-mentioned politicians’ tweets about climate change. Hence, through the access obtained by Twitter API developers’ portal, a script was written to collect all tweets containing the search words “*cambio climático*”/climate change, “*calentamiento global*”/global warming, “*efecto invernadero*”/greenhouse effect and “*medioambiente*”/environment only from the above-mentioned politicians’ Twitter profiles. The resulting *Climate Change Tweets by Spanish Politicians* corpus (CCTSP) has reached the size of 20604 tokens, including tweets posted along the period between 29-11-2010 and 28-01-2022^7^.

One of the key concepts that drive our analysis of the CCTSP corpus is that of “framing” which “essentially involves *selection* and *salience*” (Entmann, 1993: 52). It follows that keywords analysis—which is based on the notion of statistical “saliency” of lexical items in a given corpus^8^—would aid in detecting recurrent frames in climate change communication by influential Spanish politicians via Twitter. Therefore, this piece of research followed a mixed-methods approach where the quantitative keywords analysis was combined with qualitative methods, which are: manual semantic classification of keywords as well as the extraction of their concordance lines.

The tool used in the generation of keywords of the CCTSP corpus as well as the extraction of concordance lines and collocations is the *CQPweb* (Hardie, 2012). The chosen reference corpus for comparison was the *Europarl* Spanish corpus (Tiedemann, 2012),^9^ since reference corpora of “similar register will result in words very particular to the target corpus” (Hirch & Geluso, 2019: 233).

In order to compare both corpora, the *CQPweb* ran the log ratio (effect-size statistic) with log-likelihood filter (significance test) as a comparison statistic for each token of the target corpus. The tool extracted 106 positive keywords which were later filtered manually to exclude signs of punctuation, generic terms or symbols (e.g.: “@”, “http://”) as well as impertinent keywords (e.g.: “*máis*”, “é”). Later on, the final list of keywords was classified into three groups: content keywords (63), function keywords (11) and proper-nouns (12).Table 40, in the Appendices, presents the list of resulting positive keywords in the CCTSP corpus, providing the following data about each key wordform: its absolute and relative frequencies both in the target and reference corpus; its log ratio score, which represents “a doubling in size of the difference between the two corpora, for the keyword under consideration” (Hardie, 2014); and its log-likelihood value (with *p* value set to 0.0001). Each content keyword was manually assigned to its pertinent semantic category with the aid of Spanish *Multilingual Central Repository* 3.0 (MCR) database (Gonzalez-Agirre et al., 2012) (Table 41).

## Results and Discussion

In the light of all previous data that contributes to a better contextualisation of the issue of climate change in Spain on a social, political and an academic level, in this section we present the quantitative data yielded by our keyword analysis, then we carry out the qualitative classification and organization of the data in order to be able to identify recurrent themes and frames in the CCTSP corpus.

**Fig. 2:**
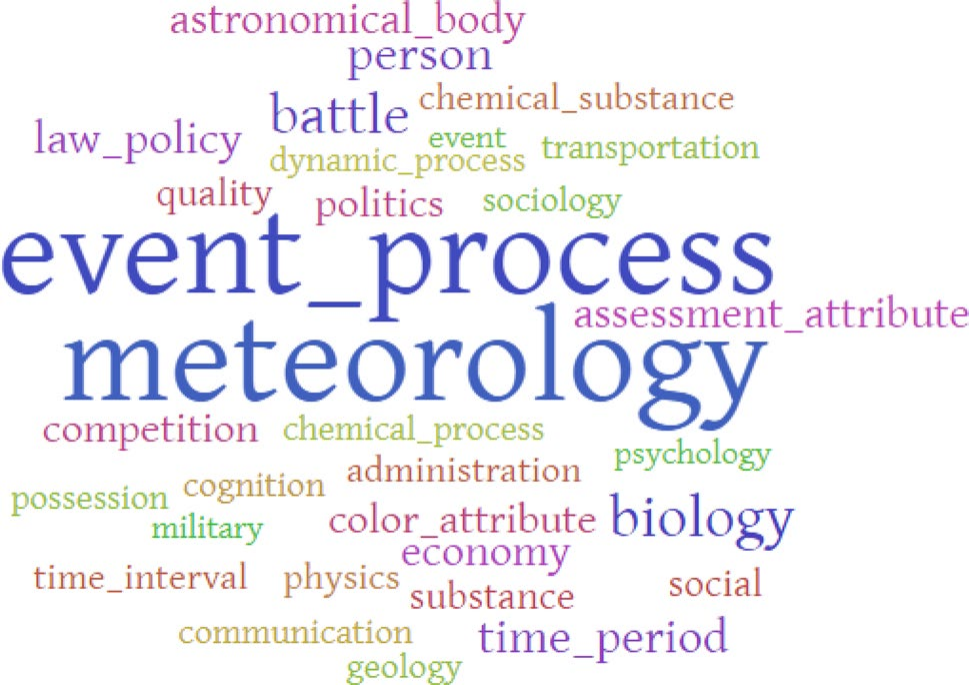
Word-cloud of semantic domains in the CCTSP corpus

Content keywords represent “robust indications of the text’s aboutness” (i.e.: its recurrent or salient themes and topics) (Scott, 2010: 43). Table 41, in the Appendices, shows keywords that have been classified as content words (i.e.: open-class words) along with their respective quantitative data and corresponding semantic domains. To help visualize the data obtained through the process of semantic classification of keywords, we have created the following word-cloud representing salient semantic domains in the CCTSP corpus (Fig. 2).

The previous image represents a small-scale ontology of prominent semantic domains in Spanish political tweeting about what originally constitutes a meteorological phenomenon: the long-term heating of Earth's climate system. While there are nodes (e.g.: biology, law-policy, geology and politics) that are of expected occurrence in this register, given their globally acknowledged intersection with the phenomenon under study; Fig. 2 has foregrounded some seemingly odd semantic domains, such as: battle, competition and military. Through the examination of concordances lines of the keywords composing each semantic domain, we have been able to identify a set of register-specific *leitmotifs*. In what follows, we discuss qualitative results obtained by the analysis of concordance lines of each content keyword in terms of its role in the configuration of a recurrent image or frame in the CCTSP corpus. In other words, we have organized the qualitative analysis of prevalent semantic domains and their pertinent content keywords according to their interrelation and their observed conceptual proximity and textual co-occurrence.

### Climate Change/Global Warming as a Meteorological Phenomenon

The first semantic domain in Table 2 constitutes the nucleus of our corpus, as it is comprised by two of the search terms used in scrapping the tweets that conform our corpus (see Section "Materials and Methods"). Looking through the collocates of both search terms: “*cambio climático*”/climate change and “*calentamiento*”/warming (Table 3), we have identified two main linguistic patterns that occur around meteorological keywords.

**Table 2:** The first group of related semantic domains and their corresponding keywords

Semantic domains	Keywords			
Meteorology	*climático*/climatic	*climática*/climatic	*clima*/climate	*calentamiento*/warming
Event_process	*cambio*/change	*transición*/transition		
Dynamic_process	*frenar*/curb	*avanza*/move forward		
Law policy	*sostenibilidad*/sustainability	*ley*/law

**Table 3:** Collocates of “*cambio climático*” and “*calentamiento global*”

No.	Word	Total no. in whole corpus	Expected collocate frequency	Observed collocate frequency	In no. of texts	Log ratio (filtered)
1	frenar/Stop	9	1.038	9	7	7.109
2	contra/Against	164	18.912	141	22	5.556
3	adaptación/Adaptation	6	0.692	5	3	5.261
4	combatir/Combat	16	1.845	13	9	5.055
5	lucha/Fight	92	10.609	74	18	4.979
6	luchar/To fight	22	2.537	17	10	4.705
7	efectos/Effects	8	0.923	6	5	4.525
8	frente/Front	14	1.614	8	5	3.355
9	Ley/Law	46	5.305	23	7	2.94
10	o/Or	41	4.728	16	7	2.296
11	sobre/On	59	6.804	23	10	2.293

The first pattern depicts climate change as if it were a naturally occurring disaster whose only victim is the human race. The following concordances (Table 4) illustrate examples of the demonization of climate change/global warming against the ‘benevolence’ of humans who need to either control or fight it; hence the presence of dynamic-processes and battle keywords in our corpus.

**Table 4:** Concordance lines of “*cambio climático*”, “*calentamiento global*” and “*climática*”

todo el mundo saldrán a la calle contra el	cambio climático	y para exigir un futuro más justo para todas
el	cambio climático	‘demoledor’ sobre los seres humanos
necesitamos un planeta que nos permita vivir con dignidad. Frenar el	cambio climático	nos compete a todos
Andalucía busca dar una respuesta conjunta y eficaz a el	cambio climático	para frenar el deterioro extremo de nuestro hábitat
200 millones de refugiados climáticos en 2050 si no ponemos freno al	calentamiento	global
todo nuestro apoyo a la manifestación pacífica #Rebelion7O para reclamar medidas urgentes frente a la crisis	climática	
es una gran noticia que EEUU vuelva al Acuerdo de París y a la lucha contra la crisis	climática	

Everyone will take to the streets against the	Climate change	And to demand a fairer future for all
The ‘devastating’	Climate change	On human beings
We need a planet that allows us to live with dignity. curbing	Climate change	Is up to all of us
Andalusia seeks to give a joint and effective response to	Climate change	To stop the extreme deterioration of our habitat
200 million climate refugees by 2050 if we don't stop	Global warming	
All our support for the peaceful demonstration #Rebelion7O demanding urgent measures against the	Climate	Crisis
It is a great news that the US returns to the Paris Agreement and to the fight against the	Climate	Crisis

The tag “*demoledor*”/devastating results as key in our corpus (Table 21) because of its prominent use in the description of climate change impact in our corpus. Interestingly, though, the use of the keyword “*clima*”/climate—when not collocating with “change”—in the CCTSP corpus reflects an opposite view where Earth’s climate is portrayed as the victim in need of rescuing (Table 5).

**Table 5:** Concordance lines of “*clima*”

El New Deal Verde necesita un esfuerzo económico , porque si el	clima	fuera un banco, ya estaría rescatado
me he reunido con @ClimateEnvoy John Kerry, Enviado especial de EEUU para el	clima	hemos hablado de la próxima #COP26 y el papel de España

The Green New Deal requires an economic effort, because if the	Climate	Was a bank, it would have been rescued
I met @ClimateEnvoy John Kerry, US Special Envoy for the	Climate	We have talked about the upcoming #COP26 and the role of Spain

Thus, in the register of Spanish political tweeting about climate change, the most frequent representation of this meteorological crisis is that of a battle where humans need to fight the enemy (i.e.: global warming) and advance towards a sustainable life (Table 6).

**Table 6:** Concordance lines of "*avanzar*"

#CumbreDelClima está muy lejos de la ambición necesaria y no sirve para	avanzar	en la lucha contra el cambio climático
una #BancaPública de inversión nos ayudará a	avanzar	hacia una transición ecológica justa y eficaz
#CumbreDelClima is far from the necessary ambition and does not serve to	Move forward	In the fight against climate change
#PublicBanking for investment will help us	Move forward	To a just and effective ecological transition

The second linguistic pattern occurring around the climate change node is the framing of the crisis as a matter of policy-making. Both left- and right-wing parties take advantage of climate change tweeting to declare their disapproval of the Spanish laws and policies concerning the environment or their lack of agreement about them (Table 7). If we zoom in some of the following examples, we will find traces of strong evaluative language (highlighted in bold in Table 7) that stir feelings of fear, anger or confusion, respectively, in the public opinion: the law of climate change will possibly harm Galician fish preserves and put into risk 40,000 jobs (Casado-Blanco, 2020), the law of climate change is disappointing “far from achieving the bare minimum” (Iñigo Errejón, 2021), the law of climate change needs “amendments in its entirety” (Meritxell Batet, 2020). Some tweets refer to the dismissal of the law by specific political parties, while others declare explicitly that the government has an interest in passing the law of climate change without the consent of the parliament, which creates an atmosphere of uncertainty and suspicion about the law project.

**Table 7:** Lines of concordance of “*ley*”

PPopular @CiudadanosCs y @eajpnv prescinden de la	ley de cambio climático	, la transición energética , autoconsumo la falta de políticas públicas ensancha los beneficios de las eléctricas.
el proyecto de	ley de cambio climático	está **muy lejos de lo mínimo necesario**
una	ley	que fija solamente una reducción del 23 % de CO2 a 2030 mientras mientras las organizaciones ecologistas, jóvenes y la ciencia esperan el 55 %. exigimos rectificación
hoy he estado en una batea en la ría de Arousa y en la lonja de Ribeira para reclamar que se mantengan los fondos europeos para la pesca española , y que la	ley de cambio climático	no **perjudique** a las conserveras y depuradoras gallegas, **arriesgando** 40.000 empleos y 1.000 empresas.
comienza el #Pleno del @Congreso_ES que se reúne en sesión extraordinaria para debatir, entre otros asuntos, una enmienda **de totalidad** al proyecto de	ley de cambio climático	y transición energética .
el gobierno quiere aprobar la	ley de cambio climático	sin pasar por el pleno del congreso

Popular @CiudadanosCs and @eajpnv disregard the	climate change law	, energy transition, self-consumption, the lack of public policies increases the benefit of electric companies.
the	climate change law	project is **very far from meeting the bare minimum**
a	law	that sets only a 23% CO2 reduction by 2030 while environmental organizations, youth and science expect 55%. we demand rectification
Today I have been in a raft in the Arousa estuary and in the Ribeira fish market to demand that the European funding of Spanish fishing be maintained, and that the	climate change law	does not **harm** Galician canning and treatment plants, by **putting into risk** 40,000 jobs and 1,000 companies
The extraordinary #Plenary session of the @Congreso_ES begins with the purpose of debating, among other issues, **full amendment** to the	law of climate change	and energy transition
The government wants to approve the	climate change law	without passing through the plenary session of the Congress

In this respect, we should add that the weight that the keywords “*transición*”/transition—that collocates exclusively with either the adjective “*ecológica*”/ecological or “*energética*”/energetic (Table 33)—acquires inside the CCTSP corpus is due to the overreference to the Spanish *Law of Climate Change and Ecological Transition*.

“*Sostenibilidad*”/Sustainability is another keyword that refers to Spanish policies regarding climate change, yet, unlike the cluster “*ley de cambio climático*/Law of climate change”, it is likely to occur in contexts of figurative language (highlighted in bold in Table 8) which reveal a tendency to regard sustainability as an abstract solution to the problem rather than a well-defined policy or an action plan (Table 9).

**Table 8:** Concordance lines of “*sostenibilidad*”

queremos que nuestra tierra sea **referencia** en la	sostenibilidad	y el medioambiente
**levantemos la bandera** de la	sostenibilidad	y el respeto por la naturaleza
he presentado en la #COP25 la #RevoluciónVerde, un compromiso sin precedentes de la región europea más vulnerable, Andalucía, que **liga su desarrollo** a	sostenibilidad	
hoy más que nunca queremos que #Andalucía sea **la capital** de la	sostenibilidad	

We want our land to be **a reference in**	Sustainability	And the evironment
Let's **raise the flag of**	Sustainability	And respect for nature
I have presented the #GreenRevolution at #COP25, an unprecedented commitment from the most vulnerable European region, Andalusia, which **links its development to**	Sustainability	
Today more than ever we want #Andalusia to be **the capital of**	Sustainability

**Table 9:** The second group of semantic domains and their corresponding keywords

Semantic domains	Keywords			
Biology	*medioambiente*/Environment	*biodiversidad*/ Biodiversity	*ecológica*/ecological	*ambiental*/ Environmental
Economy	*economía*/ Economy	*consumo*/ Consumption	*circular*/ Circular	
Politics	*compromiso*/ Commitment			
Administration	*global*/ Global	*globales*/Global		
Geology	*Costas*/ Coasts			
Cognition	*modelo*/ Model			
Astronomical_body	*planeta*/ Planet			
Time_interval	*vida*/Life			

### The Environment as an Endangered Body

On Twitter, Spanish politicians regard ecosystems, biodiversity and the environment as an unattended body which falls completely under humans’ control. Collocates of biological keywords (Table 10) insist over the framing of nature as well as the whole planet as a human possession.

**Table 10:** Collocates of biology and astronomical_body keywords

No.	Word	Total no. in Whole corpus	Expected collocate frequency	Observed collocate frequency	In no. of texts	Log Ratio (filtered)
1	Proteger/Protect	17	0.881	11	4	5.068
2	Cuidar/Take care of	10	0.518	6	3	4.778
3	Transición/Transition	37	1.918	21	7	4.585
4	Defensa/Defence	12	0.622	6	3	4.193
5	Protección/Protection	19	0.985	7	3	3.416
6	Nuestro/Our	64	3.317	14	7	2.357

Five of these collocates inculcate the metaphor of nature as an endangered body that belongs to the human race (hence, the overuse of *nuestro*/our as a collocate) that is represented as the only saviour capable of caring for/defending/protecting it. In order to grasp how this metaphor has been construed in political speeches along the years, it can be contrasted with anti-anthropocentric approaches to environmental protection.

In what follows, we examine framings of human activity (highlighted in bold in Table 11) in the pursuit of recovering nature, life and the planet from the threat of the global warming crisis. The next table of concordances (Table 11) provides examples of the use of biology keywords as well as the keywords: “*planeta*” and “*vida*”. All of the examples depict climate change as the villain that threatens human possessions and existence on Earth, and thus, humans fight back to save biodiversity, the environment, ecological units, life and the planet. All of these keywords turn to be semantically equivalent as they refer to natural resources falling completely under human will and control.

**Table 11:** Concordance lines of "*biodiversidad*", "*ecológica*", "*medioambiente*", "*planeta*" and "*vida*"

**gestionar** de manera responsable *nuestro* patrimonio común , el agua , los suelos y la	biodiversidad	es ineludible .
hoy **me he reunido** con las organizaciones ecologistas @greenpeace_esp, @ecologistas, @AmigosTierraEsp, @WWFespana y @SEO_BirdLife para compartir las líneas de trabajo sobre el impacto ecológico del consumo y medida para **combatir** el cambio climático y la pérdida de	biodiversidad	
urge **trabajar** para lograr la adaptación climática e **impulsar** la recuperación	ecológica	también en los países más vulnerables al cambio climático .
hoy **avanzamos** en transición	ecológica	el proyecto de ley de cambio climático entra en su recta final
es fundamental **concienciar** desde la infancia en el **cuidado** por el	medioambiente	y un **mayor control** **y esfuerzo** de las administraciones y de la sociedad
la voz de #Andalucía se escucha en Europa para **proteger** el	medioambiente	
seguimos apostando por **fomentar** la concienciación sobre la necesidad de **proteger** *nuestro*	planeta	
está en juego *nuestro*	planeta	#LaEspanaQueQuieresEsEcologista
el cambio climático está provocando transformaciones tales que la	vida	está severamente amenazada .
La especulación es una amenaza para las ciudades, como lo es el cambio climático para el planeta. O **priorizamos** la	vida	o **permitimos** la especulación . Nosotros **elegimos** la vida

Responsible **management** of *our* common heritage, water, soil and	Biodiversity	is inescapable
Today **I have met** with the environmental organizations @greenpeace_esp, @ecologistas, @AmigosTierraEsp, @WWFespana and @SEO_BirdLife to share the lines of work on the ecological impact of consumption and measures to **combat** climate change and the loss of	Biodiversity	
It is urgent to **work** on achieving climate adaptation and **promoting**	Ecological	Recovery, also in the countries most vulnerable to climate change.
today **we move forward** in	Ecological	Transition. The climate change bill enters its final stretch
It is essential to **raise awareness** from childhood about **caring** for the	Environment	and for **greater control and effort** from administrations and society
the voice of #Andalusia is heard in Europe to **protect** the	Environment	
We continue to bet on **promoting** awareness of the need to **protect** *our*	Planet	
*our*	Planet	Is at stake. #LaEspanaQueQuieresEsEcologista
Climate change is causing such transformations that	Life	has become severely threatened
Speculation is a threat to cities, as is climate change to the planet. Either we **prioritize**	Life	Or we **allow** speculation. we **choose** life

Regarding possible action pathways in the fight against climate change, our semantic classification of salient keywords in the CCTSP corpus point to specific fields of action toward the solution of the problem, which are listed in Table 9. This corpus’ register was dominated by the perception of this global catastrophe as a matter of geological management. For almost a decade (2010–2021), the only Spanish legislation that figured as the enacted policy to face the climate change problem was the Spanish coastal law: *Ley de costas*.^10^ The climate change and energy transition law has only been recently approved by the Spanish parliament, on the 20th of May 2021.

In parallel, the solution to global warming was, also, framed as lying within the ecopolitical sphere. Keywords referring to economic practices (Table 9) occur frequently and prominently in our corpus yet their concordance plots are unbalanced, meaning that each Spanish politician would stress over one particular economic solution over the others. For example, Juan Manuel Moreno, president of the Autonomous Government of Andalusia (2019 till current), advocates for circular economy whereas the Spanish Minister of Consumer Affairs, Alberto Garzón Espinosa (2020 till current), insists on consumption reduction as the principal strategy to reduce the effects of climate change.

Lexical cues of political solutions to climate change in our corpus rely on the overuse of the keyword “*compromiso*”/commitment whose concordances (Table 12) reveal how climate change crisis do play a vital role in political ethos representation. Spanish politicians rival to confirm and to convince with better commitment from their part to the environment and to offering a solution to the crisis.

**Table 12:** Concordance lines of "*compromiso*"

Andalucía ha pasado a ser **referencia** de	compromiso	con el medioambiente.
se cierra una cumbre del @g20org intensa y exitosa, en la que hemos (...), **reafirmado** el	compromiso	con la lucha contra el cambio climático
constatamos una vez más que son muchas las prioridades compartidas : un **firme**	compromiso	contra el cambio climático
nuestro	compromiso	es **total** y **absoluto** ; son muchos los proyectos que impulsamos para luchar contra el cambio climático
ante los grandes desafíos globales, desde el cambio climático a los movimientos migratorios o el terrorismo, debemos trabajar juntos con un	compromiso	**inequívoco** con el multilateralismo.
la lucha contra el cambio climático es un	compromiso	**personal** y **de Gobierno**
y no es solo un deseo, sino un	compromiso	**que avanza** : vamos a compartir con Galicia la vicepresidencia la mesa del @EU_CoR.

Andalusia has become a **benchmark for**	Commitment	To the environment
an intense and successful @g20org summit closes, in which we have (...), **reaffirmed** our	Commitment	To the fight against climate change
Once again we confirm that there are many shared priorities: a **firm**	Commitment	Against climate change
our	Commitment	Is **complete** and **absolute**; There are many projects that we promote to fight climate change
Faced with the great global challenges, from climate change to migration or terrorism, we must work together with an **unequivocal**	Commitment	To multilateralism.
The fight against climate change is both a **personal** and a **governmental**	Commitment	
and it is not just a wish, but a	Commitment	That is **moving forward**: we are going to share the vice-presidency of the @EU_CoR table with Galicia.

The word “*compromiso*”/commitment is often surrounded by up-scaling devices and vocabulary of positive appreciation (e.g.: solid commitment, complete and absolute commitment, unequivocal commitment, etc.) to present the authorial voice as worthy of leadership in the battle against climate change.

Despite the fact that “*global*”/global has been one of the search keywords used to collect our corpus (in “*calentamiento global/*global warming”), concordance lines of this lemma proved that its saliency in our corpus is not exclusively linked to “global warming” (which accounts for almost 20% of all its concordances). Other contexts of use of this keyword exhibit an opposite stance adopted by the authorial voice which backgrounds its political persona. The authorial voice chooses to stress on the “global” dimension of the problem almost exclusively when it needs to promote alliances with foreign countries or entities (highlighted in bold in Table 13). The individual, local or regional efforts are downsized in these particular contexts where external relations and foreign affairs are foregrounded, such as in the following examples:

**Table 13:** Concordance lines of "global"

los progresistas sabemos que para hacer frente a desafíos	globales	como el #CambioClimático, se necesitan **herramientas multilaterales**.
el #CambioClimático no se detiene, es una realidad ya presente y una amenaza para nuestro futuro. Hacerle frente requiere del esfuerzo	global	**de gobiernos e instituciones y del compromiso de todos y todas**
con el #medioambiente y el territorio . https://t.co/vqVXzeRKBh ante los grandes desafíos	globales	desde el cambio climático a los movimientos migratorios o el terrorismo, debemos trabajar juntos con un compromiso inequívoco con el **multilateralismo**.
**#COP25** , una reunión decisiva para elevar la ambición en la lucha	global	contra la emergencia climática . por responsabilidad con las generaciones presentes y

We, progressives, know that in order to face	Global	Challenges, such as #ClimateChange, **multilateral tools** are needed.
#ClimateChange does not stop, it is an already present reality and a threat to our future. Facing it requires the	Global	Effort of **governments and institutions as well as the commitment of all**
with the #environment and the territory. https://t.co/vqVXzeRKBh Facing great	Global	Challenges, from climate change to migration or terrorism, we must work together with an unequivocal commitment to **multilateralism.**
**#COP25**, a decisive meeting to raise ambition in the	Global	Fight against the climate emergency. for responsibility with present generations and

Lastly, cognitive approaches toward the solution are labelled by the keyword “*modelo*”/model which is taken to refer to the Spanish governmental planning and strategies in economy, education, food or energy sectors. In all of its hits in our corpus (21), the meaning of change is either implied or explicitly stated around this keyword (Table 14).

**Table 14:** Concordance lines of "*modelo*"

tenemos q **cambiar** nosotras y nosotros : hábitos de consumo, prioridades y	modelo	económico
el actual	modelo	de alimentación permite producir ocultando costes sociales , sanitarios y ecológicos . Es hora de **pasar a la acción**
Andalucía está **cambiando** de	modelo	y avanza en políticas de sostenibilidad
estamos poniendo la base de un futuro sostenible y** un nuevo**	modelo	económico más verde
hace falta actuar y **cambiar** nuestro	modelo	de producción y consume
es necesario **implantar **un	modelo	energético que preserve nuestro derecho al medio ambiente

We have to **change:** consumption habits, priorities and economic	Model	
The current food	Model	makes it possible to produce while hiding social, sanitary and ecological costs. It's time to** take an action**
Andalusia is **changing** its	MODEL	and making progress in sustainability policies
we are laying the foundation for a sustainable future and a **new,** greener economic	model	
we need to act and **change** our	Model	of production and consumption
it is necessary to **develop** an energy	Model	that preserves our right to the environment

### Climate Change as a War

Influential Spanish leaders often opt to rely on the “Climate Change As A War” metaphor when tweeting about their goals or achievements regarding the management of global warming (Table 15). Concordances of the keyword “luch*/fight”^11^ (Table16) usually foregrounds the role of either a part of the Spanish territories, albeit a city, a region, a province; or the whole country (highlighted in red in Table 16) as a frontliner in the “war” against climate change. The keyword “*lucha*”/fight usually occurs with either up-scaling devices (e.g.: *más*/most, *mejor defiende*/best defend), martial vocabulary (e.g.: the keyword: *vanguardia*/forefront) or expressions of positive inscribed judgement. Relatively fewer concordances of luch* deal with the “war against climate change” as a global cause with humanity as its principal protagonist.

**Table 15:** Third group of semantic domains and their corresponding keywords

Semantic domains	Keywords			
Battle	*lucha*/fight	*luchar*/fight		
Competition	*apostamos*/we bet	*apuesta/bet*	*combatir*/combat	*proteger*/protect
Military	*vanguardia*/forefront			
Sociology	*feminismo*/feminism	*desigualdad*/ inequality

**Table 16:** Concordance lines of “*lucha*”

Barcelona es un **ejemplo internacional** en la	lucha	contra la crisis climática
somos precisamente las ciudades quienes **más estamos haciendo** en la	lucha	contra el cambio climático y la contaminación
España tiene que estar **a la cabeza de la**	lucha	contra el cambio climático
España debe tener estabilidad y ser un referente en Europa con un gobierno progresista de coalición que **sea pionero** en políticas feministas	lucha	contra el cambio climático
Andalucía quiere **liderar** la	lucha	contra el cambio climático y apuesta por la economía verde
somos el partido que **mejor defiende** el medioambiente y puso a España **a la vanguardia de la**	lucha	contra el cambio climático.

Barcelona is an **international example** in the	Fight	Against the climate crisis
we are precisely the cities that are **doing the most** in the	Fight	Against climate change and pollution
Spain must be **at the forefront** of the	Fight	Against climate change
Spain must have stability and become a benchmark in Europe with a progressive coalition government that shall be a **pioneer** in feminist policies and the	Fight	Against climate change
Andalusia wants to **lead** the	Fight	Against climate change and it commits to green economy
We are the party that **best defends** the environment and puts Spain **at the forefront** of the	Fight	Against climate change

Contexts of use of this particular keyword reflect the following repetitive pattern: the authorial voice portrays itself as the hero or the saviour who urges the community that it represents to admit its views as being the only way towards salvation. This sense of urgency is usually realized through the use of modal verbs of obligation (highlighted in bold in Table 17) instead of the use of the imperative which appears to occur solely in contexts where the authorial voice chooses to address opposing political parties, to foreground their incompetence and their lack of responsibility (see second line in Table 17). In this regard, Martin & White (2005: 111) explain the shift in the authorial voice positioning from the imperative to the use of modal verbs, stating that:

The imperative is monoglossic in that it neither references, nor allows for the possibility of, alternative actions. The modal, in contrast, explicitly grounds the demand in the subjectivity of the speaker—as an assessment by the speaker of obligation rather than as a command.

**Table 17:** Concordance lines of “luchar” (1)

la furtura ley europea del clima **debe** ser un elemento central en la	lucha	contra el cambio climático
somos precisamente las ciudades quienes más estamos haciendo en la	lucha	contra el cambio climático y la contaminación. **Basta de** demagogia, señores del PP, **asuman** responsabilidades
que rodeará todo Madrid. las ciudades **debemos** liderar la	lucha	contra la contaminación y el cambio climático
**debemos** defender la seguridad energética y	luchar	contra el cambio climático : descarbonización economía y seguridad energética

The future European climate law **must be** a central element in the	Fight	Against climate change
We are precisely the cities that are doing the most in the	Fight	Against climate change and pollution. **Enough** demagogy, pp gentlemen, **assume** responsibilities
That will surround all of Madrid. Our cities **must** lead the	Fight	Against pollution and climate change
We **must** defend energy security and	Fight	Climate change : decarbonization economy and energy security

It follows that the battle against global warming takes the form of a competition among those who are in charge to prove themselves as the first winner, in the race against this global phenomenon. Concordances of competition keywords (i.e.: *combatir*/combat, *proteger*/protect, *apostar*/bet on or commit to) show a repeated representation of abstract entities in the form of either potential gains or useful weapons (highlighted in bold in Table 18) in the hands of the authorial voice who battles against the villain: the climate change. Likewise, competition keywords tend to co-occur with modal verbs of obligation (highlighted in bold in Table 18) and the collectivization of social actors^12^ performing the role of the agentive subject (e.g.: *los liberales*/liberals).

**Table 18:** Concordance lines of competition keywords

los liberales	apostamos	por **invertir más en ciencia e innovación** para reducir las emisiones sin renunciar al progreso y el bienestar
en Ramacastañas	apostamos	por **la industria maderera sostenible** con el medioambiente de Gredos
los efectos del cambio climático y la sequía son serios. **hay que**	apostar	por** la investigación**
Andalucía quiere liderar la lucha contra el cambio climático y	apuesta	por **la economía verde**
Para	combatir	eficazmente el cambio climático **solo hay una opción política viable** ponernos a trabajar urgentemente siguiendo las recomendaciones científicas
**hay que**	combatir	el cambio climático **sin disparar el precio de la energía ni destruir empleo**
**la ventana de oportunidad de**	combatir	el cambio climático
Para	proteger	el planeta, **nuestra salud** y **nuestra economía local** reduciendo las emisiones de gases de efecto invernadero y mejorando nuestro patrón de consumo, es recomendable comer de temporada
	proteger	el planeta es proteger **la economía** y **la vida**.

We, liberals,	Support	**Investing more in science and innovation** in order to reduce emissions without giving up progress and well-being
In Ramacastañas we are	Committed	To **sustainable wood industry** with the environment of Gredos
The effects of climate change and drought are serious. We **ought to**	Commit	To **research**
Andalusia wants to lead the fight against climate change and it	Commits	To **green economy**
To effectively	Combat	Climate change **there is only one viable political option**: getting to work urgently following scientific recommendations
We **must**	Combat	Climate change **without shooting up the price of energy or destroying jobs**
**The window of opportunity** to	Combat	Climate change
To	Protect	The planet, **our health** and **our local economy** by reducing greenhouse gas emissions and improving our consumption pattern, it is advisable to eat in season
	protecting	The planet is protecting **the economy** and **life**.

Another salient feature detected through the analysis of concordances of the key node “*lucha contra el cambio climático*/fight against the climate change” is the overuse of the coordinating conjunction “*y*”/and which has, in fact, resulted as one of this cluster’s collocates scoring a log ratio value of 1.26. The next table of concordances (Table 19) illustrates the coupling of “climate change”—which is quintessentially an ecological issue—with other socio-economic threats pressuring the Spanish people (e.g.: “*brecha salarial”*/pay gap, “*igualdad”*/gender equality). Thus, in the discourse of the CCTSP corpus, there exists a tendency towards an intentional pairing between climate change and other sociological issues that are framed to seem as of equivalent magnitude (Levin, 1964).

**Table 19:** Concordance lines of "luchar" (2)

compromiso con el cambio climático,	lucha	contra la brecha salarial** y** no tributar en un paraíso fiscal
Una herramienta que impulsa un uso racional del suelo, la	lucha	contra el cambio climático, la movilidad sostenible **y** el acceso a la vivienda
Europa camina en la buena dirección en cambio climático, mercado único o	lucha	contra el racismo **y** la xenofobia.
los Bienes Públicos Globales como los sistemas de salud,	luchar	contra el cambio climático, impulsar la digitalización **y** avanzar hacia la igualdad

commitment to climate change,	Fighting	Against the wage gap **and** not paying taxes in a tax haven
A tool that promotes a rational use of land, the	Fight	Against climate change, sustainable transport **and** access to housing
Europe is heading in the right direction regarding climate change, the single market or the	Fight	Against racism **and** xenophobia.
Global Public Goods such as health systems,	Fighting	Climate change, promoting digitization **and** advancing towards equality

That is why, sociology has resulted as one of the principal semantic domains of the CCTSP corpus. Sociological keywords are used exclusively in contexts of juxtaposition between environmental issues and sociological problems, particularly that of inequality and gender discrimination. In fact, it is a globally acknowledged trend to portray the feminine gender as the true victim of the global warming disaster and as the only potential saviour, because:

[w]omen commonly face higher risks and greater burdens from the impacts of climate change in situations of poverty, and the majority of the world’s poor are women. Women’s unequal participation in decision-making processes and labour markets compound inequalities and often prevent women from fully contributing to climate-related planning, policy-making and implementation. (United Nations, Climate change, UNFCC 2019)

In the CCTSP corpus, concordance lines of “*desigualdad*”/inequality and “*feminismo*”/feminism reveal traces of ecofeminism through the framing of climate change as correlating to gender inequality (Table 20).

**Table 20:** Concordance lines of sociology keywords

A.Botella : negacionista del cambio climático que escribe cuentos defendiendo la	desigualdad	de hombres y mujeres
constitución es un acuerdo de convivencia y con las generaciones anteriores y por venir. está hoy amenazado por la	desigualdad	y el cambio climático.
en el debate de presupuestos las únicas líneas rojas que ponemos son la	desigualdad	y el cambio climático
Hoy, Día mundial de la salud, es un buen momento para recordar la importancia que tienen la	desigualdad	y el deterioro ecológico en la salud
siempre valiente, inconformista, luchadora y comprometida con las causas sociales, el cambio climático y el	feminismo	
la juventud de este país está planteando con contundencia, tanto con el	feminismo	como contra el cambio climático
combatir el cambio climático es luchar por la vida y el	feminismo	

A.Botella: climate change denier who writes stories defending	Inequality	Between men and women
Constitution is an agreement of coexistence with previous and future generations. It is today threatened by	Inequality	And climate change.
In the budget debate the only red lines we draw are that of	Inequality	And climate change.
Today, World Health Day, is a good time to remember the impact of	Inequality	And ecological deterioration on health
Always brave, non-conformist, fighter and committed to social causes, climate change and	Feminism	
The youth of this country is forcefully raising the matters of both	Feminism	And climate change
Fighting climate change is fighting for life and	Feminism	

The first line of concordance (Table 20) demonstrates how this pairing holds true in the negative sense too, meaning that those who deny climate change are also defenders of inequality among men and women.

### Labelling

In the CCTSP corpus, there exists a set of attributes’ categories (Table 21) that collect *labelling* (Jeffries, 2017) tokens. Colour_attribute domain is comprised solely by the colour “*verde*”/green which is world-widely taken to symbolize biosystems and nature. This suggests that political discourse on Twitter conceives the climate change disaster as an issue related to nature rather than a man-made problem. What supports this observation is the lack of mention of other colours, like: red, yellow or blue, which are associated with global warming representation as evidenced in Mahony and Hulme (2012). Throughout the past decade, not only do Spanish politicians choose to colour their discourse about climate change and global warming in ‘green’, they also take ‘going green’ as a competition arena. The authorial voice is usually represented as ‘going green’-er^13^ than the rivals (i.e.: other political leaders) who are occasionally accused of ‘greenwashing’ (e.g.: see fourth and fifth lines in Table 22).

**Table 21:** Fourth group of semantic domains and their corresponding keywords

Semantic domains	Keywords				
Colour_attribute	*verde*/green				
Assessment_attribute	*juntos*/together	*reto*/ challenge	*retos*/ challenges		*demoledor*/ devastating
Quality	*sostenible*/ sustainable				
Psychology	*urge*/it is urgent	*negacionismo*/ denialism

**Table 22:** Concordance lines of "*verde*"

hoy hemos presentado #PlanV, un proyecto para que Madrid lidere la transición ecológica en nuestro país y asegure a los madrileños y las madrileñas un futuro **más**	verde	y más justo.
A partir de mayo Madrid va a dar un paso al frente y va a liderar la transición a un futuro más justo y **más**	verde	
es una de las prioridades del gobierno de España, avanzar hacia un planeta **más**	verde	.
basta ya mirar como si no pudiera hacerse nada, basta ya de postureo	verde	. no hay prioridad mayor .
por mucho que la izquierda se disfrace de	verde	, el liderazgo medioambiental está en @populares

Today we have presented #PlanV, a project for Madrid to lead the ecological transition in our country and ensure a	Green**er**	And fairer future for the people of Madrid
Starting in May, Madrid will take a step forward and will lead the transition to a fairer and	Green**er**	Future
It is one of the priorities of the government of Spain, moving towards a	Green**er**	Planet
Enough of looking as if nothing could be done, enough of the	Green	Posturing. There is no higher priority.
as much as the left disguises itself in	Green	, Environmental leadership is in @populares

All other kinds of attributes contribute to sketch the ‘climate change as a villain/monster’ frame, as this global disaster gets often labelled by “*reto*”/challenge or threat whose contexts usually portray the authorial voice as the hero leading the battle against climate change for the addressees to follow (hence, the overuse of the keyword “*juntos*”/together in our corpus) (Table 21). The solution to the problem is often represented by a couple of labels that are world-widely known to be associated with the preservation of the environment: “*verde*”/green and “*sostenible*”/sustainable. The following table of concordances (Table 23) shows that colour, assessment and quality attributes tend to co-occur within the same context.

**Table 23:** Concordance lines of attributes keywords

pido a todos que me ayudéis y sigamos sumando juntos a este	reto	. queremos que nuestra tierra sea referencia en la sostenibilidad
no hay	reto	más grande : sin transición verde no hay futuro posible
metiendo el hombro juntos por el medioambiente y un progreso	sostenible	donde no nos falte lo principal : el agua.
levantemos la bandera de la sostenibilidad y el respeto por la naturaleza.	juntos	seremos capaces de llegar más lejos.

I ask you all to help me and that we continue adding to this	Challenge	Together. We want our land to be a reference in sustainability
There is no bigger	Challenge	: Without green transition there is no possible future
Working together for the environment and	Sustainable	Progress as long as we do not lose the main thing: water.
Let's raise the flag of sustainability and respect for nature.	Together	We will be able to go further.

The semantic domain of psychology (Fig. 2) also gathers keywords that display an attitude through putting a label to describe affect and judgement. Those who do not believe in climate change are negatively represented, not in terms of their unscientific reasoning but in terms of their rather disordered or unsound mindset. Furthermore, the co-text surrounding the keyword “*negacionismo*”/denial inspire emotions of fear (e.g.: *más peligrosa*/more dangerous) and anger (e.g.: *su negacionismo mata*/their denialism kills) towards the actions of those who deny the phenomenon of climate change (Table 24).

**Table 24:** Concordance lines of "*negacionismo*"

PSOE e ICV estudian presentar al TC un recurso contra la ley de costas la reforma de la Ley de Costas. un salto del	negacionismo	al **autismo climático**.
hay un tipo de actitud ante el medio ambiente **tanto más peligrosa** que el puro	negacionismo	climático : hablar mucho de transición ecológica y no actuar **en absoluto**
las olas de calor por el cambio climático y las olas de la pandemia tienen algo en común : **su**	negacionismo	**mata**

PSOE and ICV are considering presenting an appeal to the TC against coastal law the reform of Coastal Law. a leap from	Denialism	To **climate autism**
There is a type of attitude towards the environment that is **even more dangerous** than pure climate	Denial	: Talking a lot about ecological transition and **not** acting **at all**
Heat waves due to climate change and pandemic waves have something in common: their	Denial	**Kills**

The keyword “*urge*”/to urge or press, on the other hand, sheds light on a side of the psychological profile of the addresser who labels his/her proposition as a compelling desire or demand. Interestingly, the contexts where this verb occurs show a more vulnerable addresser (protagonist) who usually solicits assistance from more powerful bodies (e.g.: the EU or the government of Spain). Perhaps a look-back to the classics may elucidate the repeated scene depicted in concordance lines of the verb *urge* (Table 25), as each line represents a narrative with a hero, a setting, a plot and a moral of the story.

**Table 25:** Concordance lines of "*urge*"

el cambio climático es una emergencia.	urge	actuar y buscar soluciones. Hoy, en Almería, firmamos el protocolo de actuación #Ecomares para mantener limpias nuestras costas
participo en la #PreCop26Rome, junto al presidente del @Senadoesp, @Ander_Gil.	urge	una acción ambiciosa y coordinada de la #UE
hoy siento orgullo de ver a la juventud movilizada sus consignas y carteles dicen verdades : el problema climático es un problema del sistema económico. ¡ hay que actuar ya !	urge	transformar el sistema para poner la vida , el planeta y la humanidad en el centro .
	urge	actuar contra el cambio climático. La humanidad no debe seguir jugando a la ruleta rusa.

Climate change is an emergency.	It is urgent	To act and seek solutions. Today, in Almería, we sign the #Ecomares action protocol to keep our coasts clean
I participate in the #precop26rome, together with the president of @Senadoesp, @Ander_Gil. Ambitious and coordinated #EU action is	Urgently	Needed
Today I am proud to see the youth mobilized their slogans and posters tell the truth: the climate problem is a problem of the economic system. We must act now!	It is urgent	To transform the system to put life, the planet and humanity at the center.
	It is urgent	To act against climate change. Humanity must no longer play Russian roulette.

The common element shared by all these narratives is the foregrounding of the *helper*’s role which is described in the following extract from *The Morphology of Folktale*:

if the agent received [i.e.: the helper] is a living creature, its help is directly put to use on the command of the hero. With this the hero outwardly loses all significance; he himself does nothing, while his helper accomplishes everything. The morphological significance of the hero is nevertheless very great, since his intentions create the axis of the narrative. These intentions appear in the form of various commands which the hero gives to his helpers. (Propp, 2003: 50)

Zooming in into the narratives configured in the previous examples (Table 25), we will find that in the first line the authorial voice (i.e.: the hero) resorts to European funds^14^ in order to solve the problem of coastline contamination. In the second line, the authorial voice calls and waits for the help from the European Union, while in the third, the helper takes the form of youth protests against politicians’ inaction. Finally, in the fourth line the sense of urgency foregrounds the vulnerability and incapacity of the authorial voice who condemns humanity for its passivity towards the problem.

### Climate Change as a Matter of Social Interaction

This section begins with describing prominent social actors in the CCTSP corpus’ discourse, then we proceed to explore salient social processes in the corpus, and lastly, we examine communicative events that are distinctive of the present corpus’ discourse (Table 26). The semantic category “person” includes keywords that categorize social actors who are likely to occur in Spanish political tweeting about climate change. By categorization of social actors, we mean the reference to salient groups of participants in our corpus’ discourse. For instance, the analysed tweets featured only two demonyms which are: people of Madrid and people of Andalusia. While the latter can be dismissed due to its unbalanced occurrence along our corpus (it occurred only in Juan Manuel Moreno’s tweets); the former, despite being of low frequency, has obtained a relatively high log ratio value (51.11).

**Table 26:** Fifth group of semantic domains and their corresponding keywords

Semantic domains	Keywords			
Person	*madrileños*/ people of madrid	*andaluz*/ analusian	*ecologistas*/ ecologists	*gobierno*/government
Social	*cuida*/ take care	*cuidar*/ take care	*impulsar*/promote	*liderar/* lead
communication	*enhorabuena*/ congratulations	*entrevista*/ interview		

Not only does the CCTSP corpus’ register allow for the classification of victims of this global crisis according to their home-town (e.g.: the first two concordance lines in the previous table reveal an extra care towards “*la salud de los madrileños*”/People of Madrid health), it also tends to overly-represent the capital as the leader in the war against climate change in Spain (Table 27).

**Table 27:** Concordance lines of "*madrileños*"

Los trabajadores defienden un servicio público esencial para la movilidad, el medioambiente y la salud de los	madrileños	. les acompañamos en sus reivindicaciones
tenemos un compromiso con nuestro pacto de Gobierno, con el medioambiente y con la salud de los	madrileños	
AlmeidaPP es ejemplo de buen gobierno, el alcalde de todos los	madrileños	sin sectarismo, ni revisionismo. Madrid es la prueba del éxito de sociedades abiertas, líder en bajada de impuestos, atracción de turismo, inversión internacional, movilidad y cuidado del medioambiente
un proyecto para que Madrid lidere la transición ecológica en nuestro país y que asegure a los	madrileños	y las madrileñas un futuro más verde

The workers defend a public service which is essential for mobility, the environment and the health of	People of Madrid	. we support your demands
We have a commitment to our governmental pact, to the environment and to the health of the	People of Madrid	
AlmeidaPP is an example of good government, the mayor of all	people of MADRID	Without sectarianism or revisionism. Madrid is the proof of success of open societies, a leader in lowering taxes, attracting tourism, international investment, mobility and caring for the environment
A project for Madrid to lead the ecological transition in our country and to ensure a greener future for the	People of Madrid	

Likewise, patterns of use of professionally categorized social actors (i.e.: “*ecologistas*”/ecologists and “*gobierno*”/government) reveal traces of the centralist approach^15^ ingrained in Spanish politicians’ worldview communicated on Twitter. Firstly, the relatively few references to the role of ecologists (log ratio value of 31.87) in a corpus about climate change and the environment, gives an idea of the ‘marginalisation’ of this group when compared to political leaders and/or members of the government as the log ratio score of “*gobierno*” is 2669.38. Secondly, the lexical patterning around this token shows an intentional backgrounding of ecologists who are depicted as a powerless group that is incapable of taking an action. In all three tweets, the authorial voice is foregrounded as the sole decision maker who chooses to listen to ecologists (e.g.: see third line of Table 28) or to inform of their views through external attribution^16^ (see first and second lines of Table 28).

**Table 28:** Concordance lines of "*ecologistas*"

una ley que fija solamente una reducción del 23 % de CO2 a 2030 mientras las organizaciones	ecologistas	, jóvenes y la ciencia esperan el 55 %. exigimos rectificación
pedimos a la Ley de Cambio Climático ni más ni menos que lo que **piden** las organizaciones	ecologistas	. más ambición o no contará con el voto verde.
hoy me he reunido con las organizaciones	ecologistas	, @ecologistas, @AmigosTierraEsp, @WWFespana y @SEO_BirdLife para compartir las líneas de trabajo sobre el impacto ecológico del del consumo y medidas para combatir el cambio climático y la pérdida de biodiversidad

a law that sets only a 23% reduction in CO2 by 2030 while	Environmental	Organizations, youth and science expect 55%. We demand rectification
We ask the Climate Change Law neither more nor less than what the	Environmental	Organizations ask for. More ambition or it will not get the green vote.
Today I have met with the	Environmental	Organizations, @ecologistas, @amigostierraesp, @wwfespana and @SEO_birdlife to share the lines of work on the ecological impact of consumption and on measures to combat climate change and the loss of biodiversity

On the other hand, concordances of the keyword “*gobierno*” depict the central government in charge as the only entity having the upper hand in setting policies regarding how to handle this global disaster. When talking about governmental action towards the management of climate change on Twitter, influential Spanish politicians limit their discourse to praise or attack the government in charge, using mostly vocabulary of either positive or negative judgement or appreciation (highlighted in bold in Table 29).

**Table 29:** Concordance lines of "*gobierno*" (1)

AlmeidaPP es ejemplo de **buen**	gobierno	, el alcalde de todos los madrileños sin sectarismo, ni revisionismo
FF a @sanchezcastejon por el repaso que le ha hecho al ministro Cañete en el congreso sobre el **desprecio** del	gobierno	al cambio climático la ley de costas ‘ privatiza de facto el litoral ’
estos meses hemos demostrado ser un	gobierno	**con ambición**.
gobernar es procurar el **bien**. este	Gobierno	**lo** ha demostrado estos 10 meses trabajando en los retos que España tiene por delante

AlmeidaPP is an example of **good**	Government	, The mayor of all people of madrid without sectarianism or revisionism
FF to @sanchezcastejon for the review he has made to Minister Cañete in the congress about the	Government's	**Disregard** of climate change the coastal law ' privatizes de facto the coastline '
these months we have shown that we are a	Government	**With ambition**.
To rule is to pursue what is **good.** This	Government	Has demonstrated **this** the past 10 months by working on the challenges that spain faces

Another distinctive pattern in the contexts of use of “*gobierno*” is the foregrounding of central or local governmental achievements employing linguistic devices of regional or political classification (e.g.: the genitive construction: X *de* Y) around the key node “*gobierno*”/government (Table 30). This centralist approach also leaves its mark on the configuration of leadership perception in the CCTSP corpus. In all of its occurrences, “*liderar*”/to lead takes, uniquely, as a subject, entities that refer to central or regional governments: “*Madrid debe liderar*”/Madrid must lead, “*Andalucía quiere liderar*”/Andalusia wants to lead, “*España tiene que liderar*”/Spain has to lead…

**Table 30:** Table of concordances of "*gobierno*" (2)

La lucha contra el cambio climático es un compromiso **firme** del	gobierno	**de Andalucía**. tenemos una gran oportunidad para liderar una transición ecológica modélica, sostenible y generadora de prosperidad,
el	Gobierno	**de CyL** apuesta por la sostenibilidad y la lucha contra el cambio climático
es una de las prioridades del	gobierno	**de España**, avanzar hacia un planeta más verde.
el cambio climático pasa de ser una " profecía maya " a uno de los ejes de la propuesta de	gobierno	**de Rajoy**
el proyecto del	gobierno	**socialista** asume los desafíos que tiene este país como la educación, el retorno del talento o el cambio climático
el	gobierno	**socialista** paraliza el trasvase Tajo-Segura por motivos ideológicos. Y encima pone de excusa el Mar Menor el día en el que el gobierno **regional del PP** aprueba un decreto positivo para el turismo, agricultura y medioambiente

The fight against climate change is a firm commitment of the **Andalusian**	Government	. We have a great opportunity to lead an exemplary, sustainable ecological transition that generates prosperity.
The	Government	**Of cyl** is committed to sustainability and the fight against climate change
It is one of the priorities of the	Government	**Of spain**, moving towards a greener planet.
Climate change goes from being a "Mayan prophecy" to one of the axes of the proposal of** Rajoy's**	Government	
The project of the **socialist**	Government	Assumes the challenges that this country has such as education, the return of talent or climate change
The **socialist**	Government	Paralyzes the tajo-segura water transfer for ideological reasons. And on top of that, it uses the mar menor as an excuse, meanwhile on the same day the **pp regional government** approves a positive decree for tourism, agriculture and the environment

The other distinctive social processes in our corpus display actions of solidarity and social participation: *cuidar-cuida*/protect(s), *impulsar*/boost or promote. These verbs turn to reinforce the frame of the environment, planet and human life as an endangered body that needs a serious intervention by the saviour (see section 3.2). Contexts of use of both keywords (Table 31) reflect a setting that is built upon on one or more of the following elements: (1) giving praise or support (i.e.: showing positive affect) (see third line), (2) expressing urgency by means of deontic modals (see fifth line), and (3) foregrounding financial resources (see fourth line).

**Table 31:** Concordance lines of social keywords

la #RevoluciónVerde funciona y	cuida	el medioambiente
la #RevoluciónVerde avanza con soluciones, consenso y una gestión más sostenible. os animo a	cuidar	cada gota para que gane #Andalucía y el medioambiente.
Todo **mi reconociemiento** a los efectivos del @plan_INFOCA por su **magnífico** trabajo (...) **gracias** por	cuidar	de nuestro medioambiente
" **más inversiones** para crear conciencia y	cuidar	el medioambiente con la #RevoluciónVerde :
los fondos europeos **deben** servir también para reorientar la economía hacia la mejora de la salud,	cuidar	el medioambiente y aumentar la productividad
hoy en #Washington he trasladado a @el_BID que es un socio fundamental para	impulsar	la agenda sobre el cambio climático en América Latina y que España puede apoyar con su experiencia a dotar a la región de las necesarias infraestructuras sostenibles y resilientes
el plan Madrid 360 va a ser galardonado por la Unesco por	impulsar	la movilidad eléctrica y facilitar un entorno urbano sostenible con el medioambiente.

The #greenrevolution works and	Takes care	Of the environment
The #greenrevolution moves forward by providing solutions, consensus and a more sustainable management. I encourage you to	Take care	Of every drop so that #andalusia and the environment win.
**All my appreciation** to the staff of @plan_INFOCA for their **magnificent** work (...) **Thank you** for	Taking care	Of our environment
"**More investments** to raise awareness and	Care	For the environment with the #greenrevolution:
European funds **must** also help to reorient the economy towards improving health,	Caring	For the environment and increasing productivity
Today in #Washington I have transfered to @el_BID which is a fundamental partner in	Promoting	The agenda on climate change in latin america and which spain can support with its experience to provide the region with the necessary sustainable and resilient infrastructures
The plan Madrid 360 is going to be awarded by the Unesco for	Promoting	Electric mobility and facilitating an environmentally sustainable urban environment.

According to Martin and White (2005) both the use of deontic modals and the first-person plural possessive (e.g.: “*nuestro*”/our in the third line of Table 31) constitute useful linguistic resources to achieve engagement and better alignment with the addressees as well as the contraction of dialogistic alternatives in order to prepare the putative reader to accept the views being communicated as the only valid option. Regarding the deployment of the financial frame in social keywords’ contexts, this observation supports evidence from previous research like that of Shanahan (2007: 2) who points out that: “the ‘money’ frame will chime with politicians and the private sector” in his review of media coverage of climate change in the UK.

The social dimension in the CCTSP corpus is manifested too in communication keywords, which allude to preferred moulds of social interaction inside this particular register. While it is only logical to find communication vocabulary in a corpus collected from a social media platform; the keywords “*enhorabuena*”/congratulations and “*entrevista*”/interview are not the typical communication vocabulary that is of expected recurrence in other media registers like press discourse. For example, in their study of English press discourse, Jeffries and Walker (2017: 30) state that their keywords analysis generated the following communication wordforms: *say*, *admitted*, *insisted* and *revealed*. In contrast, our corpus is rich in felicitations and video sharing of television interviews that reflect a rather positive over-representation of the self by means of evaluative expressions of affect, judgement or appreciation^17^ (highlighted in bold in Table 32).

**Table 32:** Concordance lines of communication keywords

que **de verdad importan** ( aunque respondemos a todo lo que nos pregunten ? ? ). os dejo mi	entrevista	en @el_pais :https://t.co/glI40mQAr7
siempre **valiente**, **inconformista**, **luchadora** y **comprometida** con las causas sociales, el cambio climático y el feminismo. muy **merecido**.	enhorabuena	, Isabel Coixet
**orgulloso** del papel que la #COP26 reconoce a España. está en **las mejores manos**.	enhorabuena	, Teresa
gracias a todo el equipo del @ICEX_ por **vuestra dedicación** a la #COP25. **muchas** horas de trabajo que han permitido situar a #Espana en el epicentro del debate mundial sobre el cambio climático	enhorabuena	

that **really matter** ( although we have answered everything they asked ? ). Here is my	Interview	with @el_pais: https://t.co/glI40mQAr7
always **brave, non-conformist**, **fighter** and **committed** to social causes, climate change and feminism. much **deserved**.	Congratulations	, Isabel Coixet
**proud** of the role of #COP26 in acknowledging Spain. **You are in the best hands.**	Congratulations	, Theresa
Thank you to the entire @ICEX_ team for **your dedication** to #COP25. **many** hours of work that have made it possible to place #Spain at the epicenter of the global debate on climate change	Congratulations	

Our findings go in line with previous research that detects heavier self-centred tendencies in political communication on social media platforms (Papacharissi, 2010: 238), which is manifested in the overrepresentation of self-image. Of quite relevance, too, is the statistical analysis carried out by Kristinsdottir et al. (2021) that demonstrates a positive statistical correlation between narcissism and the frequent use of social media platforms, especially Twitter. Their analysis gives an explanation to the possible reasons behind the prominence of exchanging positive feedback on Twitter and sharing content that involves great deal of self-representation (as for example, videos of broadcast interviews in the case of CCTSP corpus):

individuals high on communal narcissism value power and grandiosity in a communal domain, by seeking admiration for being a “saint”. Individuals high on communal narcissism rate themselves high on traits such as altruism, benevolence and warmth towards others, but are extremely driven by the need to validate power. (Kristinsdottir et al., 2021: 2)

### Causes and Consequences of Climate Change

These categories are grouped together because they include keywords that refer to causes and consequences of climate change as viewed and communicated in the CCTSP corpus (Table 33).

**Table 33:** Sixth group of semantic domains and their corresponding keywords

Semantic domains	Keywords		
Substance	*gases*/gases	*agua*/water	
Chemical_substances	*co2*/CO_2_		
Events	*incendios*/ fires		
Physics	*calor*/heat	*energética*/energetic	
Chemical_process	*emisiones*/ emissions		
Transportation	*movilidad*/ transportation		

Carbon dioxide emissions, which is the principal cause of the climate change disaster, occur in the CCTSP corpus 1.6 times per 1000 words (Table 34). The contexts where one of these four keywords: “*CO2*”, “*gases*”, “*emisiones*”, “*efecto invernadero*”, are employed usually to express disappointment with set policies regarding gas emissions. Vocabulary of explicit negative affect, appreciation and judgement can be found in almost all of the previous keywords’ contexts:

**Table 34:** Concordance lines of “*CO2*”, “*emisiones*” and “*gases*”

la crisis económica tapa una **crisis mucho más gorda** : el cambio climático. sigue aumentando la emisión de	CO2	. el panel internacional sobre el cambio climático adelanta un océano ártico sin hielo en el verano de 2030. **una catástrofe.**
PE vota en contra de estabilizar el mercado de emisiones	CO2	. Nuevo **golpe** al combate contra el cambio climático . **Lamentable .**
comparativa de reducción de emisiones de	CO2	en 2030 . **juzguen** ustedes mismos : Alemania : 55 Francia : 40 % Reino Unido : 68 % España : 23 %
los aviones vuelan en las capas altas de la atmósfera y generan	emisiones	de efecto invernadero **muy peligrosas** para el planeta . En la cumbre
en 2018 hubo un nuevo récord de	gases	de efecto invernadero en la atmósfera . España , en **grave riesgo** de desertificación.

the economic crisis covers up a **much bigger crisis**: climate change.	CO2	Emission continues to increase. The international panel on climate change predicts an ice-free arctic ocean by the summer of 2030. **A catastrophe**.
PE votes against stabilizing	CO2	Emissions market. New **blow** to the fight against climate change. **Pitiful.**
comparasion of	CO2	Emissions reduction in 2030. **Judge** for yourself: germany: 55 france: 40% united kingdom: 68% spain: 23%
Planes fly in the upper layers of the atmosphere and generate greenhouse	Emissions	That are **very dangerous** to the planet. At the summit
in 2018 there was a **new record** of greenhouse	Gases	In the atmosphere. Spain, at **serious risk** of desertification

Given that the transport sector in Spain is the main culprit of global warming in Spain (see section 1.3), the collected tweets forming the CCTSP corpus underrepresents this topic, in terms of frequencies and lexical diversity since the only keyword that was found to be pertaining to the semantic category of transportation was “*movilidad*”/transportation which occurs once every 1000 words. In this respect, it has been pointed out that the Spanish press, during the first decade of the XXI century, deliberately ignored discussing transportation as the reason behind climate change (Chávez, 2013; Hernández et al., 2016).

On another note, direct current consequences of global warming in Spain, such as “*calor*”/heat and “*incendios*”/wildfires occur 0.2 times and 0.6 times per 1000 words, respectively. Occurrences of both keywords correlate with vocabulary of negative affect and appreciation which is accompanied, sometimes, with up-scaling devices (e.g.: “*intenso*”/intense) (Table 35).

**Table 35:** Concordance lines of "*calor*" and "*incendios*"

Europa alcanza **máximos históricos** de	calor	. el cambio climático nos recuerda que no existe plan B
todo mi reconocimiento a los efectivos del @Plan_INFOCA por su magnífico trabajo para combatir el fuego en estos días de **intenso**	calor	. tras una noche** dura**
una **histórica **ola de	calor	**azota **el sureste de Europa , desatando cientos de incendios que **destruyen todo** a su paso
las olas de	calor	por el cambio climático y las olas de la pandemia tienen algo en común : su negacionismo **mata**
el calentamiento global también repercute en los	incendios	: **mayor voracidad** y frecuencia
si no actuamos ya el cambio climático nos traerá	incendios	**cada vez peores**
el medioambiente en la Comunitat Valenciana tiene dos grandes **enemigos** : los	incendios	y el Consell

Europe reaches **hisortical records** of	Heat	. Climate change reminds us that there is no plan b
all my appreciation to the staff of @Plan_INFOCA for their magnificent work to fight the fire in these days of **intense**	Heat	. After a **hard** night
A **historic**	Heat	Wave **hits** southeast europe, sparking hundreds of fires that **destroy everything** in their path
	Heat	Waves due to climate change and pandemic waves have something in common: their denial kills
global warming also affects	Fires	: **Greater voracity** and frequency
if we do not act now, climate change will bring us **worse and worse**	fires	
The environment in the Valencian Community has two great **enemies:**	fires	And the *consell*

Regarding water problems caused by global warming in Spain, they do get a relatively higher attention than the heat and wildfires, as the keyword “*agua*”/water has a normalised frequency of 1 per 1000 words. Qualitative analysis of this keyword’s contexts shows that influential Spanish politicians tend to focus on the following three axes when discussing water problems: (1) water scarcity (see first and second lines), (2) water pollution (see third line), and (3) water conservation (see fourth line) (Table 36).

**Table 36:** Concordance lines of "*agua*"

el cambio climático no solo se combate en Nueva York . también hay que construir infraestructuras de	agua	frente a la sequía y las inundaciones.
el 30 % de la superficie española es crítica en materia de	agua	por el cambio climático
la ganadería extensiva , social y familiar , contaminan el suelo y	agua	, contribuyen decisivamente al cambio climático y condenan al mundo rural
3 millones de lechugas sin recoger , 120 millones de litros de	agua	desperdiciados . ante el cambio climático los gestos individuales son anecdóticos si no se acompañan de una política racional sobre el uso del agua.

Climate change is not only fought in New York. it is also necessary to build	Water	infrastructures against drought and floods.
30% of the Spanish surface area is critical in terms of	Water	Resources due to climate change
Extensive farming, on a social and on a family level, contaminates the soil and	Water	, Which definitely contributes to climate change and puts the blame on rural areas
3 million lettuces not collected, 120 million liters of	Water	Wasted. Facing climate change, individual gestures are anecdotal if they are not accompanied by a rational policy on the use of water.

### Temporalization of Climate Change

In the present corpus’ register, discussing climate change is often realized around specific time frames referring to the present moment of the speech or the near future (Tables 37, 38). The only two wordforms that have resulted as collocating with “*hoy*”/today are the auxiliary verbs *he* and *hemos* conjugated with the first-person pronoun, singular and plural. This reinforces previous findings suggesting the overrepresentation of self-image by foregrounding recent actions or daily contributions of the authorial voice regarding the matter of climate change (see section 3.5).

**Table 37:** Time-period semantic domain and its corresponding keywords

Semantic domains	Keywords		
Time-period	*hoy*/today	*2021*	*2030*

**Table 38:** Concordance lines of "*hoy*"

frente al cambio climático nosotros tenemos un plan .	Hoy	**hemos** **presentado** #PlanV , un proyecto para que Madrid lidere la transición ecológica en nuestro país.
	Hoy	**nos hemos comprometido** a situar la cuestión del cambio climático como un objetivo central de nuestra actuación
	Hoy	**he compartido** con @BarackObama un interesante intercambio de ideas sobre la lucha contra el calentamiento global
	Hoy	**he** **asistido **a la manifestación por un tren que vertebre el territorio y enfríe el planeta, dentro de una estrategia eficaz y contundente contra el cambio climático y la crisis ecosocial.

Facing climate change we have a plan.	Today	**We have presented** #PlanV, a project for Madrid to lead The ecological transition in our country.
	Today	**We have committed** to placing the issue of climate change as a central objective of our action
	Today	**I have shared** with @BarackObama an interesting exchange of ideas on the fight against global warming
	Today	**I have attended** the demonstration for a train that will give structure to the territory and cool the planet, within an effective and conclusive strategy against climate change and the ecosocial crisis.

Previous recent studies have already pointed out that climate change communication in the Spanish press peaks around international global warming events, like climate change conferences (Fernández-Reyes, 2010; Fernández-Reyes et al., 2015). Similarly, our corpus data also alludes to more frequent tweeting about the issue around the years 2019 and 2021 (Table 39), which are the dates corresponding to the organization of COP25 held in Madrid, and the passing of the climate change law in Spain, respectively.

**Table 39:** Publication years of the CCTSP corpus' tweets

Publication date (year)	Number of tweets
January 2022	18
2021	225
2020	126
2019	315
2018	95
2017	80
2016	27
2015	30
2014	75
2013	80
2012	29
2011	27
2010	1

## Conclusions

Through computer assisted discourse (CAD) analysis of a decade of Spanish politicians’ tweets about climate change/global warming, we have been able to identify a set of distinctive images, themes and frames that are susceptible of shaping the public perception of the issue. In this section, we summarize key findings of the analysis.

The metaphors that pervade Spanish politicians’ communication of climate change on Twitter are those representing the human race as in war against climate change, and thus, climate change is portrayed as the villain, as if it were a natural disaster rather than human-caused. The main culprit of climate change, which is gas emissions, completely absents the ‘war against climate change’ metaphors’ contexts.

Among the thematic components that have been found to permeate our corpus’ discourse is the reference to sustainability, ecological transition and environmentally friendly (i.e.: green) lifestyles as key abstract solutions to climate change, with no further detail on ways of their implementation. Similarly, the affinity between climate change as an environmental problem and other social problems in Spain, especially gender inequality, has been repeatedly observed throughout our corpus. The centralist approach adopted by the Spanish government has also turned into one of the main themes in the CCTSP corpus, together with the heavy usage of the language of evaluation that is directed to condemn political opponents’ actions or policies and/or to praise one self’s endeavours in the war against climate change.

Finally, our mixed-methods analysis of the CCTSP corpus has contributed to uncover a systemic worldview of the global warming phenomenon as a self-occurring threat. Thus, the generalized narrative of climate change implies a political leader who takes on the role of the saviour against an enemy in a setting of a war or competition, as well as a proposed solution (i.e.: moral of the story) that excels in the battle against the overheating of Earth’s climate. Further future studies are needed to build upon these primitive results through applying more specific analytical frameworks like the Narrative Policy Framework (McBeth et al., 2014) and Appraisal Theory Framework (Martin & White, 2005) to the study of the linguistic patterns and frames detected in the present paper which constitute the first attempt towards the final goal of dismounting unworking narratives of climate change in Spain and building new narratives that lead to more effective action.

## Appendix

See Tables

**Table 40:** List of keywords of the CCTSP corpus

No.	Word	In whole “CCTSP”:		In corpus “Europarl 3: Spanish”:		+/−	Log ratio	Log likelihood	Classification
		Frequency	Frequency	Frequency	Frequency				
		(absolute)	(Per mill)	(absolute)	(Per mill)				
1	nós	41	1989.9	8	0.2	+	13.3	578.29	Function-word
2	CO2	13	630.95	4	0.1	+	12.64	178.62	Content-word
3	Rajoy	12	582.41	5	0.12	+	12.2	161.41	Proper_noun
4	madrileños	4	194.14	3	0.07	+	11.35	51.11	Content-word
5	PP	23	1116.29	19	0.47	+	11.22	291.03	Proper_noun
6	2021	4	194.14	4	0.1	+	10.94	49.58	Content-word
7	PSOE	8	388.27	8	0.2	+	10.94	99.16	Proper_noun
8	¡	10	485.34	11	0.27	+	10.8	122.61	Punctuation
9	máis	8	388.27	9	0.22	+	10.77	97.83	Portuguese
10	medioambiente	77	3737.14	95	2.35	+	10.64	931.63	Content-word
11	negacionismo	3	145.6	4	0.1	+	10.52	35.94	Content-word
12	o	41	1989.9	60	1.48	+	10.39	485.54	Function-word
13	andaluz	8	388.27	12	0.3	+	10.35	94.43	Content-word
14	primo	8	388.27	12	0.3	+	10.35	94.43	Content-word
15	da	4	194.14	8	0.2	+	9.94	45.4	Content-word
16	feminismo	4	194.14	8	0.2	+	9.94	45.4	Content-word
17	BCN	3	145.6	8	0.2	+	9.52	32.62	Proper_noun
18	ecosocial	4	194.14	12	0.3	+	9.35	42.68	Content-word
19	demoledor	3	145.6	10	0.25	+	9.2	31.46	Content-word
20	abrazo	3	145.6	10	0.25	+	9.2	31.46	Content-word
21	IPCC	3	145.6	10	0.25	+	9.2	31.46	Proper_noun
22	lidera	5	242.67	17	0.42	+	9.17	52.27	Content-word
23	2030	12	582.41	48	1.19	+	8.94	122	Content-word
24	climático	406	19704.91	1711	42.29	+	8.86	4097.71	Content-word
25	cuida	5	242.67	25	0.62	+	8.62	48.82	Content-word
26	¿	10	485.34	59	1.46	+	8.38	94.62	Punctuation
27	liderar	10	485.34	78	1.93	+	7.98	89.43	Content-word
28	apostamos	6	291.21	60	1.48	+	7.62	50.85	Content-word
29	climática	17	825.08	182	4.5	+	7.52	141.88	Content-word
30	digitalización	4	194.14	44	1.09	+	7.48	33.17	Content-word
31	os	9	436.81	110	2.72	+	7.33	72.84	Function-word
32	Andalucía	13	630.95	166	4.1	+	7.26	104.12	Proper_noun
33	EEUU	12	582.41	158	3.9	+	7.22	95.41	Proper_noun
34	granito	4	194.14	53	1.31	+	7.21	31.75	Content-word
35	Cañete	6	291.21	91	2.25	+	7.02	46.08	Proper_noun
36	cambio	398	19316.64	7871	194.53	+	6.63	2860.33	Content-word
37	planeta	52	2523.78	1041	25.73	+	6.62	371.62	Content-word
38	cuidar	10	485.34	263	6.5	+	6.22	66.17	Content-word
39	ecologistas	5	242.67	149	3.68	+	6.04	31.87	Content-word
40	Galicia	13	630.95	415	10.26	+	5.94	81.14	Proper_noun
41	Ecológica	28	1358.96	915	22.61	+	5.91	173.52	Content-word
42	entrevista	7	339.74	232	5.73	+	5.89	43.18	Content-word
43	'	12	582.41	410	10.13	+	5.84	73.32	Punctuation
44	Madrid	22	1067.75	799	19.75	+	5.76	131.83	Proper_noun
45	ganadería	6	291.21	228	5.63	+	5.69	35.42	Content-word
46	calentamiento	7	339.74	275	6.8	+	5.64	40.88	Content-word
47	calor	8	388.27	316	7.81	+	5.64	46.63	Content-word
48	París	15	728.01	595	14.71	+	5.63	87.32	Proper_noun
49	vanguardia	7	339.74	287	7.09	+	5.58	40.3	Content-word
50	urge	7	339.74	291	7.19	+	5.56	40.11	Content-word
51	Enhorabuena	11	533.88	465	11.49	+	5.54	62.68	Content-word
52	España	71	3445.93	3210	79.33	+	5.44	395.54	Proper_noun
53	costas	14	679.48	721	17.82	+	5.25	74.44	Content-word
54	circular	8	388.27	416	10.28	+	5.24	42.38	Content-word
55	apuesta	7	339.74	370	9.14	+	5.22	36.87	Content-word
56	vía	67	3251.8	3613	89.29	+	5.19	350.45	Function-word
57	NO	109	5290.23	6105	150.88	+	5.13	562.39	Function-word
58	Transición	37	1795.77	2114	52.25	+	5.1	189.36	Content-word
59	Frenar	9	436.81	558	13.79	+	4.99	44.64	Content-word
60	desigualdad	9	436.81	565	13.96	+	4.97	44.42	Content-word
61	sostenibilidad	17	825.08	1108	27.38	+	4.91	82.69	Content-word
62	invernadero	15	728.01	1002	24.76	+	4.88	72.25	Content-word
63	"	361	17520.87	24648	609.17	+	4.85	1729.37	Punctuation
64	biodiversidad	9	436.81	636	15.72	+	4.8	42.38	Content-word
65	avanza	8	388.27	571	14.11	+	4.78	37.52	Content-word
66	CO2	13	630.95	968	23.92	+	4.72	59.91	Content-word
67	verde	42	2038.44	3148	77.8	+	4.71	193.09	Content-word
68	globales	8	388.27	726	17.94	+	4.44	33.85	Content-word
69	incendios	11	533.88	1011	24.99	+	4.42	46.29	Content-word
70	reto	20	970.69	1901	46.98	+	4.37	82.89	Content-word
71	ambiental	12	582.41	1153	28.5	+	4.35	49.49	Content-word
72	gases	13	630.95	1257	31.07	+	4.34	53.46	Content-word
73	retos	18	873.62	1892	46.76	+	4.22	71.18	Content-word
74	Ley	46	2232.58	5129	126.76	+	4.14	176.86	Content-word
75	Clima	15	728.01	1819	44.96	+	4.02	55.29	Content-word
76	sostenible	41	1989.9	5214	128.86	+	3.95	147.54	Content-word
77	lucha	92	4465.15	12182	301.07	+	3.89	324.36	Content-word
78	.	18	873.62	2530	62.53	+	3.8	61.41	Punctuation
79	Movilidad	14	679.48	2067	51.09	+	3.73	46.49	Content-word
80	Energética	16	776.55	2402	59.36	+	3.71	52.64	Content-word
81	impulsar	11	533.88	1746	43.15	+	3.63	35.06	Content-word
82	?	303	14705.88	48655	1202.49	+	3.61	963.08	Punctuation
83	luchar	22	1067.75	3605	89.1	+	3.58	68.86	Content-word
84	Consumo	21	1019.22	3699	91.42	+	3.48	62.96	Content-word
85	frente	14	679.48	2530	62.53	+	3.44	41.32	Function-word
86	Agua	23	1116.29	4191	103.58	+	3.43	67.55	Content-word
87	combatir	16	776.55	3043	75.21	+	3.37	45.75	Content-word
88	juntos	12	582.41	2528	62.48	+	3.22	32.11	Content-word
89	emisiones	15	728.01	3595	88.85	+	3.03	36.72	Content-word
90	modelo	21	1019.22	5078	125.5	+	3.02	51.09	Content-word
91	contra	164	7959.62	40064	990.17	+	3.01	396.93	Function-word
92	!	25	1213.36	6497	160.57	+	2.92	57.69	Punctuation
93	Global	19	922.15	5091	125.82	+	2.87	42.84	Content-word
94	compromiso	46	2232.58	12678	313.33	+	2.83	101.52	Content-word
95	proteger	17	825.08	4786	118.28	+	2.8	36.88	Content-word
96	Gobierno	55	2669.38	19834	490.19	+	2.45	96.63	Content-word
97	:	164	7959.62	62241	1538.26	+	2.37	275.1	Punctuation
98	economía	25	1213.36	9985	246.78	+	2.3	39.78	Content-word
99	vida	27	1310.43	11749	290.37	+	2.17	39.32	Content-word
100	nuestro	64	3106.19	30492	753.6	+	2.04	84.38	Function-word
101	Hoy	54	2668.91	30833	762.03	+	1.81	58.23	Content-word
102	é	38	1878.12	24048	594.34	+	1.66	35.5	Portuguese
103	con	206	10181.39	255453	6313.43	+	0.69	40.65	Function-word
104	El	792	39143.97	1009884	24958.92	+	0.65	142.92	Function-word
105	para	249	12306.63	324594	8022.22	+	0.62	40.08	Function-word
106	.	882	43592.15	1437244	35520.97	+	0.3	35.93	Punctuation

40 and

**Table 41:** List of content keywords and semantic domains in the CCTSP corpus

No.	Word	In whole “CCTSP”:	Log Ratio	Log likelihood	Semantic domain	Reason for exclusion
		**Frequency (absolute)**				
1	CO2	13	12.64	178.62	Chemical_substance	
2	madrileños	4	11.35	51.11	person	
3	2021	4	10.94	49.58	Time_period	
4	medioambiente	77	10.64	931.63	biology	
5	negacionismo	3	10.52	35.94	Psychology	
6	andaluz	8	10.35	94.43	person	
7	primo	8	10.35	94.43		Irrelevant
8	da	4	9.94	45.4		Polysemy
9	feminismo	4	9.94	45.4	Sociology	
10	ecosocial	4	9.35	42.68		Unbalanced concordance plot
11	demoledor	3	9.2	31.46	Assessment_Attribute	
12	abrazo	3	9.2	31.46		Irrelevant
13	lidera	5	9.17	52.27		Unbalanced concordance plot
14	2030	12	8.94	122	Time_period	
15	climático	406	8.86	4097.71	Meteorology	
16	cuida	5	8.62	48.82	Social	
17	liderar	10	7.98	89.43	Social	
18	apostamos	6	7.62	50.85	Competition	
19	climática	17	7.52	141.88	Meteorology	
20	digitalización	4	7.48	33.17		Unbalanced concordance plot
21	granito	4	7.21	31.75		Irrelevant
22	cambio	398	6.63	2860.33	Event_process	
23	planeta	52	6.62	371.62	Astronomical_body	
24	cuidar	10	6.22	66.17	Social	
25	ecologistas	5	6.04	31.87	Person	
26	Ecológica	28	5.91	173.52	Biology	
27	entrevista	7	5.89	43.18	Communication	
28	ganadería	6	5.69	35.42		Unbalanced concordance plot
29	calentamiento	7	5.64	40.88	Meteorology	
30	calor	8	5.64	46.63	Physics	
31	vanguardia	7	5.58	40.3	Military	
32	urge	7	5.56	40.11	Psychology	
33	Enhorabuena	11	5.54	62.68	Communication	
34	costas	14	5.25	74.44	Geology	
35	circular	8	5.24	42.38	Economy	
36	apuesta	7	5.22	36.87	Competition	
37	Transición	37	5.1	189.36	Event_process	
38	Frenar	9	4.99	44.64	Dynamic_process	
39	desigualdad	9	4.97	44.42	Sociology	
40	sostenibilidad	17	4.91	82.69	Law_policy	
41	invernadero	15	4.88	72.25		Constituent of a multi-word Expression
42	biodiversidad	9	4.8	42.38	Biology	
43	avanza	8	4.78	37.52	Dynamic_process	
44	CO2	13	4.72	59.91	Chemical_Substance	
45	verde	42	4.71	193.09	Colour_attribute	
46	globales	8	4.44	33.85	Administration	
47	incendios	11	4.42	46.29	Event	
48	reto	20	4.37	82.89	Assessment_attribute	
49	ambiental	12	4.35	49.49	Biology	
50	gases	13	4.34	53.46	Substance	
51	retos	18	4.22	71.18	Assessment_attribute	
52	Ley	46	4.14	176.86	Law_policy	
53	Clima	15	4.02	55.29	Meteorology	
54	sostenible	41	3.95	147.54	Quality	
55	lucha	92	3.89	324.36	Battle	
56	Movilidad	14	3.73	46.49	Transportation	
57	Energética	16	3.71	52.64	Physics	
58	impulsar	11	3.63	35.06	Social	
59	luchar	22	3.58	68.86	Battle	
60	Consumo	21	3.48	62.96	Economy	
61	Agua	23	3.43	67.55	Substance	
62	combatir	16	3.37	45.75	Competition	
63	juntos	12	3.22	32.11	Assessment_attribute	
64	emisiones	15	3.03	36.72	Chemical_process	
65	modelo	21	3.02	51.09	Cognition	
66	Global	19	2.87	42.84	Administration	
67	compromiso	46	2.83	101.52	Politics	
68	proteger	17	2.8	36.88	Competition	
69	Gobierno	55	2.45	96.63	Person	
70	economía	25	2.3	39.78	Economy	
71	vida	27	2.17	39.32	Time_interval	
72	Hoy	54	1.81	58.23	Time_period	

41.

## Footnotes

1 Up to the year 2005, there were only four academic publications about climate change communication in Spain (Evriti & León, 2017: 2).

2 In this paper, we use the term “register” to refer to a text variety associated with a certain situation or a purpose (Biber, 1994: P. 32)

3 Zappavigna (2017: 203) gives a brief history of CDS research performed on corpora extracted from Twitter.

4 One of the current manifestations of the reduction of water resources in Spain is the drought suffered by the Tagus River basin in the Iberian Peninsula, which has been documented in the paper published by Sodermann & De Oliviera (2022: 1) who conclude that: “climate change will significantly change the stream flow regime and reduce water availability in the Tagus River basin”.

5 *SparkToro* tool is an online resource that measures the influence of a Twitter account by taking into account the size of the account as well as the engagement it receives (https://sparktoro.com/tools/sparkscore).

6 The latest edition of the list for 2022, together with the method of analysis and selection, is available in: https://los500.merca2.es/project/los-50-politicos-mas-influyentes-en-espana-en-2022/

7 The corpus collection process ended on Saturday, the 29^th^ of January 2022, at 20:08:44 (GMT+2).

8 In CorpLing analysis, the term “saliency” refers to the statistically significant repetition of a lexical item in a given corpus. The statistical significance is calculated by performing log-likelihood test for each token of the corpus under study, taking into account its frequency both in the target corpus and the reference corpus (Baker, 2006).

9 The *Europarl* Spanish corpus is a tagged corpus consisting of approximately 40 million words extracted from the proceedings of the European Parliament covering several topics including climate change: https://ec.europa.eu/info/topics_en.

10 The keyword “*costas*”/coasts is used to refer to Spanish coastal law in all of its occurrences in the CCTSP corpus.

11 The asterisk (*) is a regular expression that refers to any string of characters following the word-root preceding it.

12 According to van Leeuwen’s model: “The representation of social actors”, collectivization is the representation of social actors as participants of a homogenous group (van Leeuwen, 1995: 50).

13 The word “*más*”/more is the first function word that has resulted as a collocate of the keyword “*verde*”/green in the CCTSP corpus.

14 *Ecomares* project was fully funded by the European Union (EFE, 17 enero 2020).

15 Similar findings revealing strong centralist approaches in the Spanish government were observed in a recent study of the Spanish governmental response in times of the Covid-19 crisis (Navarro & Velasco, 2022).

16 Martin & White (2005: 102) define attribution as the representation of the proposition communicated by the addresser “as grounded in the subjectivity of an external voice” in order to achieve a more heteroglossic engagement with the addressees.

17 Appraisal framework developed by Martin & White (2005) gives full account of possible linguistic resources employed in expressing affect (emotion), judgement (ethics) and appreciation (aesthetics).
